# 1,2-Difunctionalizations of alkynes entailing concomitant C–C and C–N bond-forming carboamination reactions

**DOI:** 10.1039/d1ra06633a

**Published:** 2022-03-04

**Authors:** Santosh Kumar Nanda, Rosy Mallik

**Affiliations:** Department of Chemistry, School of Applied Science, Centurion University of Technology and Management Paralakhemundi Odisha-761211 India sknanda@cutm.ac.in

## Abstract

Vicinal carboamination of alkynes is a highly reliable and efficient practical strategy for the quick preparation of valuable and diverse amine derivatives starting from simple synthons. The last decade has witnessed numerous practical methods employing transition-metal-based/metal-free carboamination approaches using alkynes for the synthesis of these N-bearing entities. Driven by the renaissance of transition metal catalysis, intermolecular and intramolecular carboamination of alkynes comprising concomitant C–N and C–C bond formation has been studied extensively. In contrast to metal catalysis, though analogous metal-free approaches have been relatively less explored in the literature, they serve as alternatives to these expensive approaches. Despite this significant progress, reviews documenting such examples are sporadic; as a result, most reports of this type remained scattered throughout the literature, thereby hampering further developments in this escalating field. In this review, different conceptual approaches will be discussed and examples from the literature will be presented. Further, the reader will get insight into the mechanisms of different transformations.

## Abstract 2

The 1,2-difunctionalization of alkynes happening through concomitant C–C and C–N bond formation strategies have provide an unified access to diversely functionalized N-bearing heterocycles.

## Graphical abstract

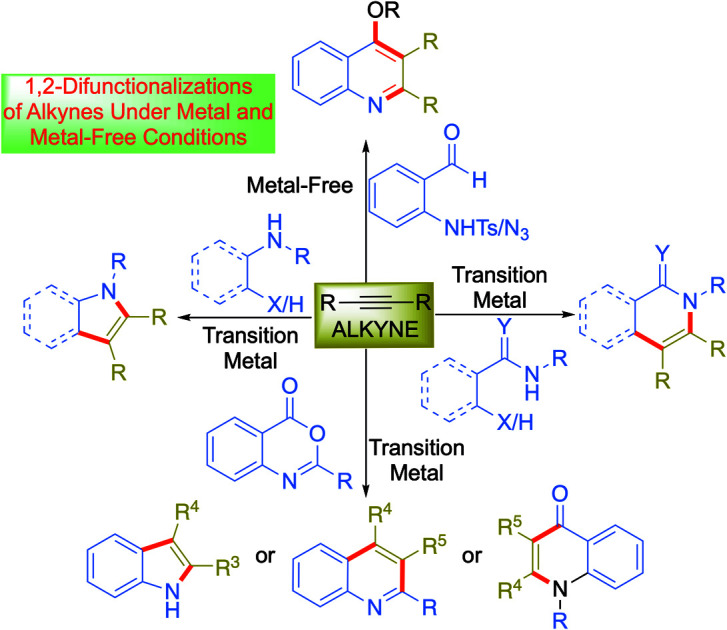

## 1. Introduction

Carbon–carbon multiple bonds are essential and versatile synthons that engage in diverse organic transformations. Difunctionalization of carbon–carbon multiple bonds by constructing two different vicinal chemical bonds is an invincible strategy and has attracted significant attention from the synthetic community in the last decade. In this context, carboamination of alkenes, alkynes, and allenes has provided a straightforward route for the synthesis of functionalized amines and their congener heterocycles.^1–12^

Carboamination of alkynes has come to the forefront as a method of choice for the synthesis of an avenue of N-bearing aromatic heterocycles and has attracted significant attention from the synthetic community.

**Fig. 1:**
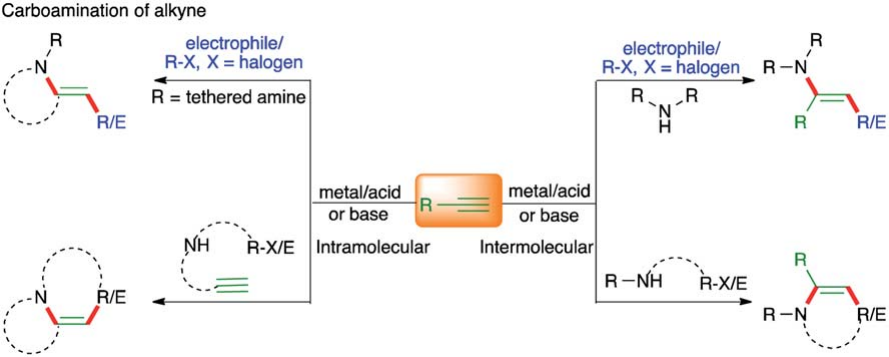
Different possible paths for the carboamination of alkynes under metal/metal-free conditions.

The other arguable advantage of this protocol may be the quick access to a synthetically useful and celebrated intermediate, *i.e.* enamine (Fig. 1). To showcase the proficiency of this method, various strategies have been developed in the literature. In this context, the contribution of transition metal catalysis has been exceptional and has delivered an enormous number of protocols for the expedient synthesis of N-bearing scaffolds. On the other hand, corresponding metal-free approaches have also been documented in the literature for the synthesis of variously substituted N-heterocycles. Unlike the gathering together of various reports on the carboamination of alkenes in the literature as reviews, the analogous compilation of literature on carboamination of alkynes is scant.

In the present review, an overview of developments on the diastereoselective as well as the enantioselective carboamination of alkynes is given, with specific attention to the mechanism for each transformation in detail. The review contains sections in which the reactions are categorized under various headings depending upon the operative reaction mechanism. The first such section will describe the developments in carboamination of alkynes using transition metal-catalyzed oxidative addition to fragments consisting of both carbon and nitrogen source. The second section will elaborate the reactions based upon metal-catalyzed C–H activation. The third section will enlighten upon reports of carboamination triggered by cycloaddition reactions. The fourth section will demonstrate carboamination of benzynes. The fifth section will tell us about the intramolecular carboamination approach towards the synthesis of complex aza-cycles. The final section will elaborate on methods utilizing electrocatalysis in carboamination reactions and metal-free carboamination reactions.

## 2. Carboamination of alkynes

### 2.1 Transition metal-catalyzed carboamination

Transition metals have shown promising reactivity in the difunctionalization of alkynes. In this context, carboamination of alkynes has been studied extensively for the synthesis of N-bearing aromatic heterocycles. Several strategies involving transition metal-catalyzed C–H bond functionalization, cycloaddition, difunctionalization of benzyne, and intramolecular carboamination have been studied. Furthermore, corresponding metal-free approaches have also been reported. This section will provide a concise idea about the recent developments.

#### 2.1.1 Oxidative addition-triggered carboamination

**Scheme 1:**
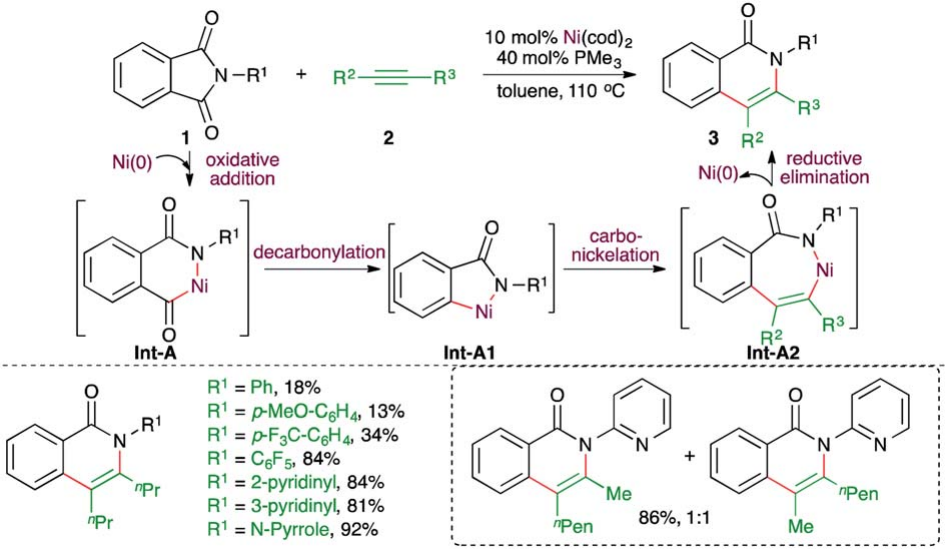
Decarbonylative carboamination reaction of phthalimides with alkynes.

In this domain, Ni-catalyzed decarbonylative carboamination reaction of phthalimides to alkynes was studied by Kurahashi and co-workers for the synthesis of isoquinolones (Scheme 1).^13^ Reaction of phthalimides 1 and alkynes 2 with 10 mol% Ni(cod)_2_ and 40 mol% PMe_3_ resulted in various isoquinolones 3 in good to excellent yield. When the substrate scope was studied, it was found that the presence of electron-withdrawing substituents on the *N*-aryl ring gave better yields in comparison with phenyl or aryl rings bearing electron-donating groups. Further, unsymmetrical alkynes led to a mixture of regio-isomers. The formation of the product can be explained as follows. Nickelacycles Int-A was formed by oxidative addition, which upon decarbonylation gave Int-A1. The Int-A1 furnished Int-A2*via* carbo-nickelation with alkynes. Finally, reductive elimination led to the formation of 3.

**Scheme 2:**
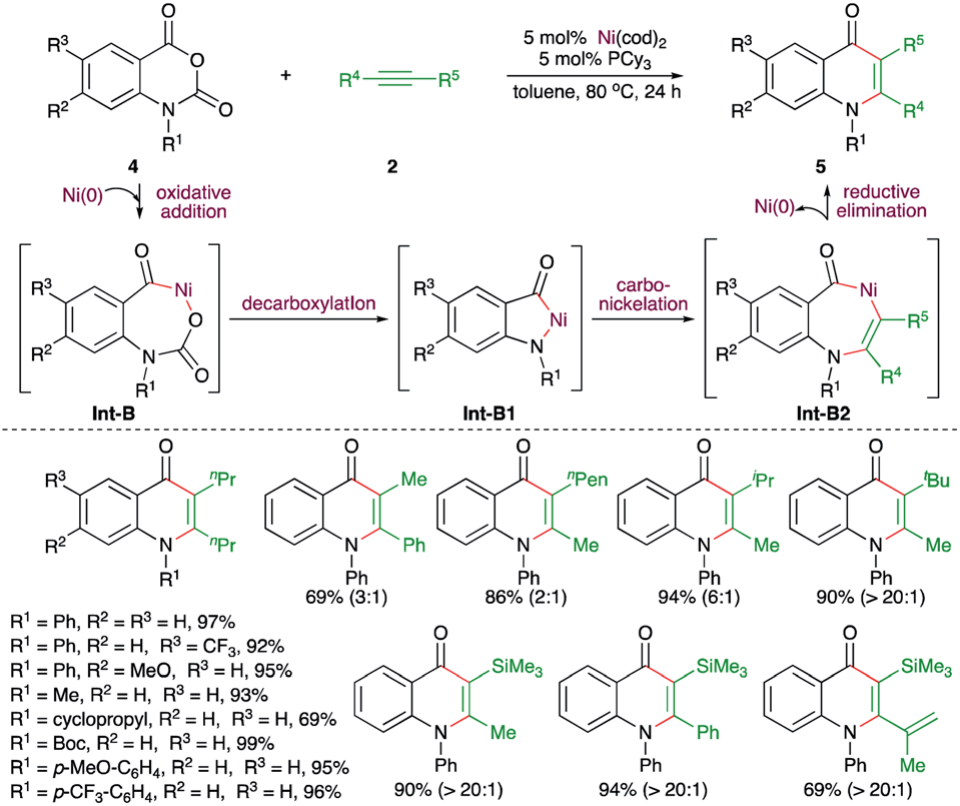
Decarboxylative carboamination reaction of phthalimides with alkynes.

On similar lines, Matsubara and co-workers have reported a Ni-catalyzed decarboxylative carboamination strategy for the facile synthesis of 4-quinolones (Scheme 2).^14^ Reaction of *N*-arylisatoic anhydrides 4 with alkynes 2 in the presence of 5 mol% Ni(cod)_2_ and 5 mol% PCy_3_ afforded the corresponding 4-quinolones 5 in good yield. Interestingly, both aliphatic and aromatic symmetrical alkynes resulted in the formation of the desired quinolone. In the case of unsymmetrical alkynes, a mixture of regio-isomers was formed. The regio-isomeric ratio was dictated by the bulkiness of the substituent attached to the alkynes. The effect of bulkiness was so pronounced that the presence of *tert*-butyl and TMS groups led to the exclusive formation of a single product with the less bulky group near to the nitrogen atom. The reaction followed a similar path to arrive at 5 through Int-B to Int-B2.

**Scheme 3:**
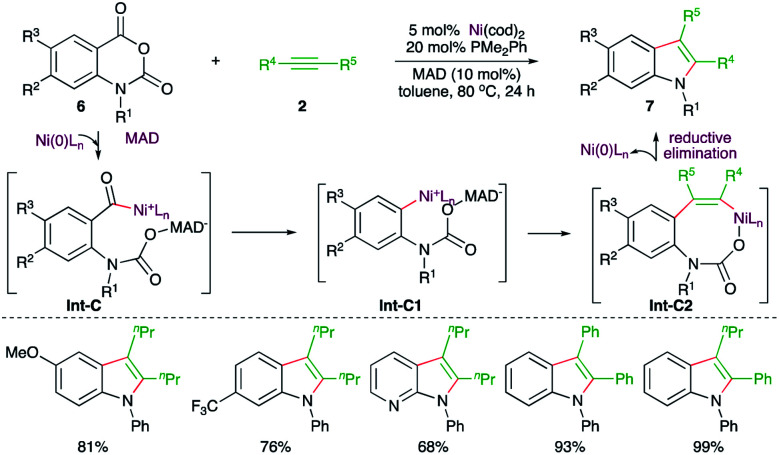
Decarbonylative–decarboxylative carboamination cascade.

Similarly, a Ni-catalyzed decarbonylative–decarboxylative carboamination cascade was reported by Matsubara and co-workers for the facile synthesis of indoles (Scheme 3).^15^ Reaction of isatoic anhydrides 6 with alkynes 2 in the presence of 5 mol% Ni(cod)_2_, 20 mol% PMe_2_Ph, and 10 mol% of the additive MAD (methylaluminum bis(2,6-di-*tert*-butyl-4-methylphenoxide)) furnished the corresponding indole derivatives 7 in good yield. The suggested mechanism revealed that the use of MAD is responsible for the formation of 7 not 5 (see Scheme 7) as it chose a different reaction pathway. Thus, in the presence of MAD, Int-C was formed, which upon decarbonylation gave Int-C1. The Int-C1 performed carbo-nickelation with alkynes to generate Int-C2. The Int-C2 then underwent decarboxylation and reductive elimination of nickel to give indoles. The entire different path may be attributed to temporary coordination of bulky DMAD to the carbamate inhibiting the decarboxylation prior to decarbonylation.

**Scheme 4:**
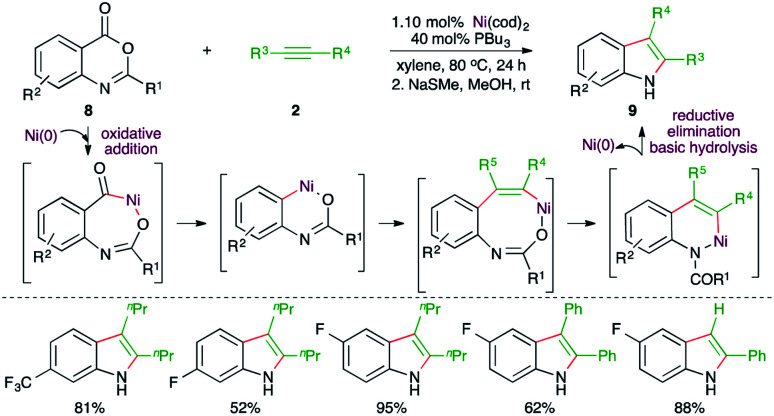
Decarbonylative–acyl migration–carboamination–amide hydrolysis cascade.

Further, Matsubara and co-workers have also reported a Ni-catalyzed decarbonylative-acyl migration–amide hydrolysis carboamination cascade for the expedient synthesis of variously substituted indoles from readily available anthranilic acid derivatives (Scheme 4).^16^ Reaction of anthranilic acid derivatives 8 with alkynes 2 in the presence of 10 mol% Ni(cod)_2_ and 40 mol% PPr_3_ furnished the corresponding indoles 9 in good to excellent yield. Initially, the N-protected amide was obtained along with some unprotected indole. Thus, after the reaction, basic hydrolysis was carried out to get rid of the protecting group. Variously substituted indoles were synthesized from different alkynes. However, regio-isomeric mixtures were obtained in the case of unsymmetrical alkynes.

**Scheme 5:**
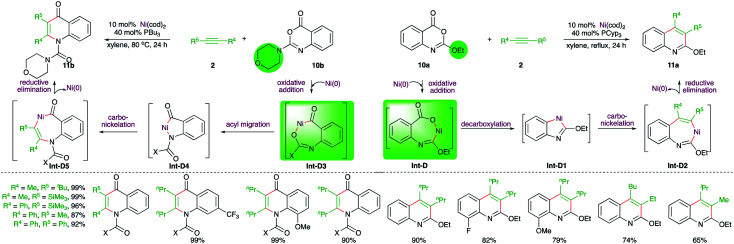
Ni-catalyzed protecting group-dependent divergent synthesis of N-heterocycles.

Matsubara and co-workers have also reported an elegant method describing the effect of protecting groups on the Ni-catalyzed intermolecular carboamination reactions. It was observed that benzoxazinones 10a with alkoxide protecting group (R^1^ = alkoxide) upon reaction with alkynes in the presence of 10 mol% Ni(cod)_2_ gave 2-alkoxyquinolines 11a, whereas the presence of amine as the protecting group in 10b (R^1^ = amine) led to the formation of quinolones 11b under the same reaction conditions (Scheme 5).^17^ The different outcome could be explained depending upon the operative mechanism. In the case of alkoxide-tethered benzoxazinones 10a, the initial oxidative addition takes place at the vinylic C–O bond guided by the offered temporary coordination from the oxygen atom to generate Int-D, which upon decarboxylation gives metallacycle Int-D1. Int-D1 upon subsequent carbo-nickelation and reductive elimination furnishes 11a*via*Int-D2. On the other hand, when the protecting group is bigger, like ^*t*^Bu or *N*-morpholinyl, the temporary coordination factor is overruled by steric prohibition of the oxidative addition at the vinylic C–O bond. Thus, the oxidative addition occurs at the carbonylic C–O bond to give Int-D3, which upon acyl migration furnishes Int-D4. The Int-D4 upon carbo-nickelation and reductive elimination affords quinolones 11b*via*Int-D5.

**Scheme 6:**
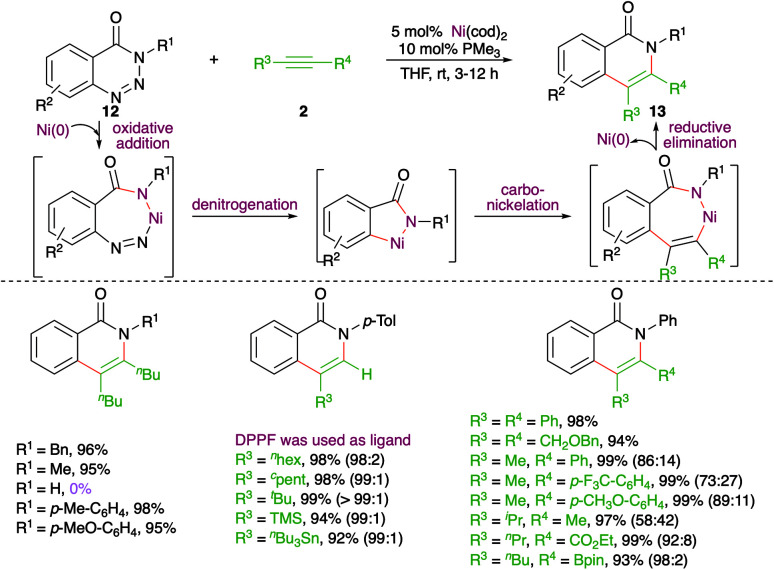
Denitrogenative carboamination cascade.

A nickel-catalyzed domino denitrogenative-carboamination strategy was developed by Murakami and co-workers for the synthesis of isoquinolones (Scheme 6).^18^ Reaction of benzotriazin-4(3*H*)-ones 12 with alkynes 2 in presence of nickel(0)/phosphine catalyst gave corresponding isoquinolones 13 in good yield. Both internal and terminal alkynes were engaged in the reaction. However, in the case of unsymmetrical alkynes and terminal alkynes, a mixture of regio-isomers was obtained and the ratio was found to be a function of steric factors. The bulkier the substituent, the higher is the selectivity.

#### 2.1.2 C–H insertion-triggered carboamination

**Scheme 7:**
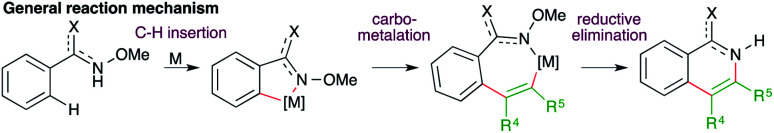
Mechanism for the carboamination of alkynes involving C–H functionalization.

During the invention of these elegant methods, another strategy became very popular for the synthesis of these N-bearing heterocycles. The strategy relied upon the directing group-assisted/free formation of metallacycle *via* insertion at C–H bonds. The metallacycle then participated in a carbometalation process with alkynes to generate a new metallacycle. Finally, reductive elimination of metal led to the formation of N-bearing heterocycles (Scheme 7).

**Scheme 8:**
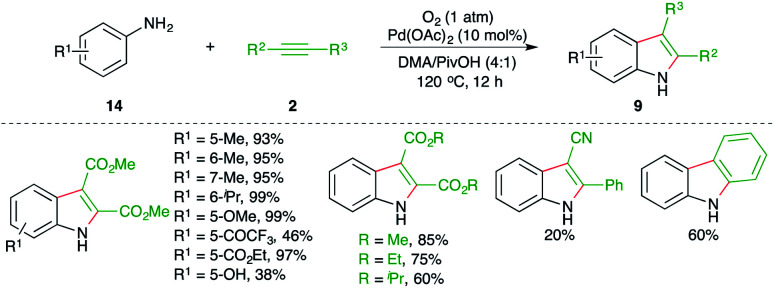
Carboamination of alkynes with anilines.

Jiao and co-workers have reported a practical synthesis of indole using Pd-catalyzed carboamination of alkynes with anilines (Scheme 8).^19^

The reaction of anilines 14 with electron-deficient alkynes 2 in the presence of 10 mol% Pd(OAc)_2_ and molecular oxygen as oxidant gave variously substituted indoles 9 in good to excellent yield. The reaction has an excellent scope and functional group compatibility. The reaction only worked in the case of electron-deficient alkynes.

**Scheme 9:**
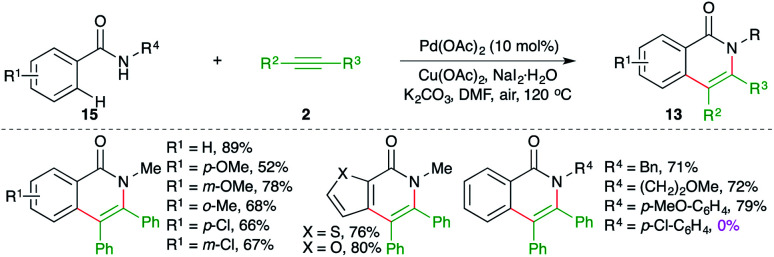
Carboamination of alkynes with aryl amides.

On similar lines, Huang and co-workers have used palladium instead of ruthenium to effect the carboamination cascade. The reaction of *N*-alkyl/aryl benzamides 15 with alkynes 2 in the presence of 10 mol% Pd(OAc)_2_ and 2 equivalents of copper acetate as oxidant delivered the corresponding *N*-aryl/alkyl iso-isoquinolones 13 in good yield (Scheme 9).^20^ The reaction demonstrated excellent scope for aryl amides. However, a limited number of alkynes were used in the transformations. Further, in the case of unsymmetrical alkynes, a mixture of regio-isomers was obtained and the regioselectivity was governed by the bulkiness of the group present on nitrogen as aryl groups led to the exclusive formation of one regio-isomer. It should be noted that *N*-aryls and halogen-substituted aryls failed to give the product.

**Scheme 10:**
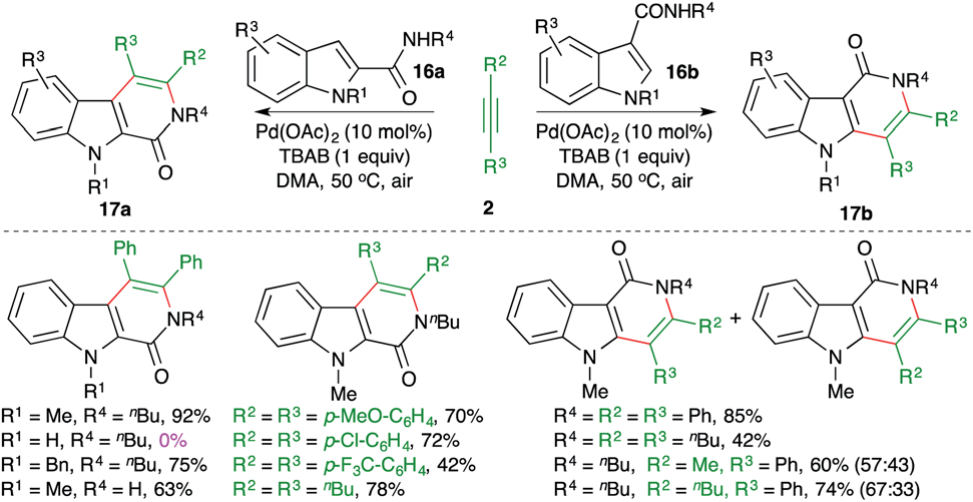
Carboamination of alkynes with indole-tethered amides.

Jiao and co-workers have reported a Pd-catalyzed intermolecular carboamination method for the rapid synthesis of β- and γ-carbolinones from the corresponding indole-tethered amides and alkynes in air (Scheme 10).^21^ The treatment of amides 16a,b with alkynes 2 in the presence of 10 mol% Pd(OAc)_2_ gave the respective carbolines 17a,b in good yield.

The reaction worked well for aliphatic and aromatic alkynes. However, in the case of unsymmetrical alkynes, a mixture of regio-isomers was obtained. Unfortunately, free indoles (R^4^ = H) did not participate in the reaction.

**Scheme 11:**
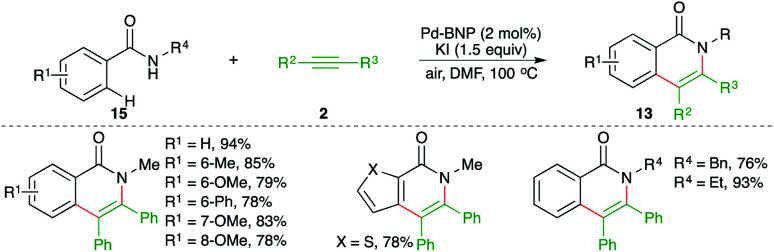
Carboamination of alkynes with amides for the synthesis of isoquinolones.

A binaphthyl-stabilized palladium nanoparticle (Pd-BNP)-catalyzed intermolecular carboamination of alkynes was reported by Sekar and co-workers for the facile synthesis of various isoquinolones in good yield. The Pd-BNP was easily recovered and could be reused four times without loss in activity. The reaction of amides 15 with alkynes 2 in the presence of 4 mol% Pd-BNP gave the corresponding isoquinolones 13 in good yield (Scheme 11).^22^ Aromatic internal alkynes were found to be the suitable substrate for coupling rather than aliphatic alkynes.

**Scheme 12:**
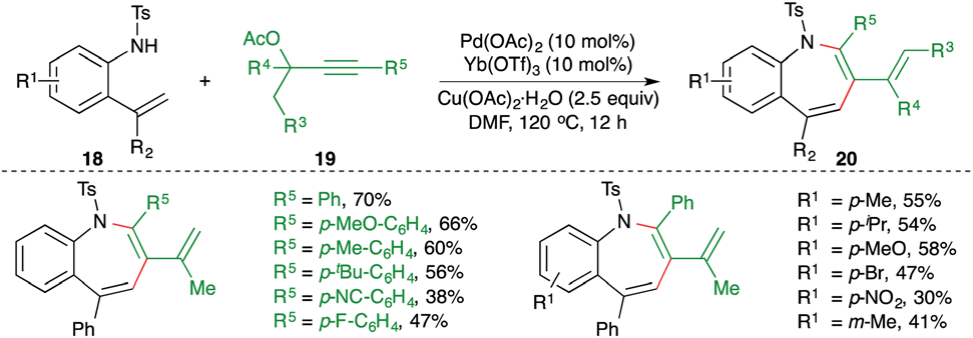
Carboamination of alkynes with *o*-alkenyl anilines.

A combined catalytic system consisting of palladium and ytterbium triflate catalyst for the carboamination reaction of 2-alkenyl anilines 18 with propargylic esters 19 was studied by Zeng and co-workers for the facile synthesis of benzo[*b*]azepine derivatives 20 (Scheme 12).^23^ Although the reaction had an excellent scope, terminal alkynes could not form the desired benzo[*b*]azepines under the optimized conditions.

**Scheme 13:**
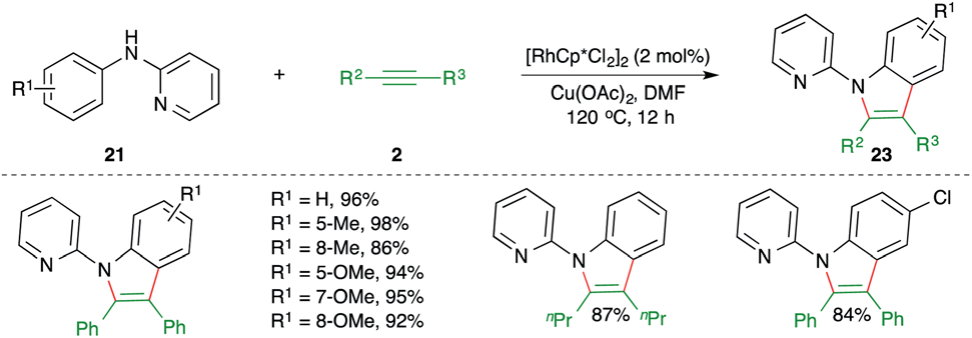
Directing group-assisted carboamination of alkynes with anilines.

On similar lines, Li and co-workers have studied Rh-catalyzed carboamination reaction of alkynes 2 with 2-pyridienyl anilines 21 for the synthesis of pyridine-tethered indoles 22 (Scheme 13).^24^ Here pyridine acted as the directing group. The method is limited to only symmetrical alkynes.

**Scheme 14:**
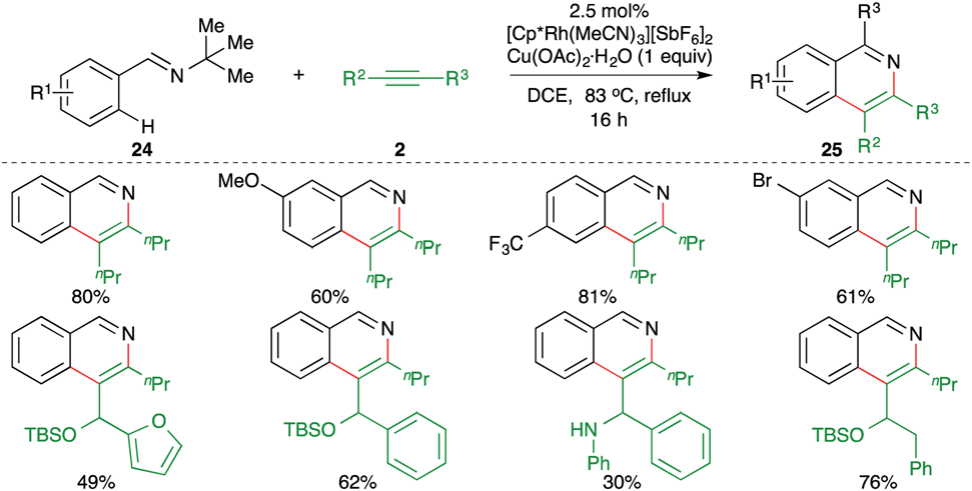
Carboamination of alkynes with imines.

A Rh-catalyzed intermolecular carboamination of aryl imines with alkynes delivered the corresponding isoquinolines, as reported by Fagnou and co-workers. The reaction of imines 24 with alkynes 2 in the presence of 2.5 mol% [Cp*Rh(MeCN)_3_][SbF_6_]_2_ formed substituted isoquinolines 25 in good to excellent yield (Scheme 14).^25^

**Scheme 15:**
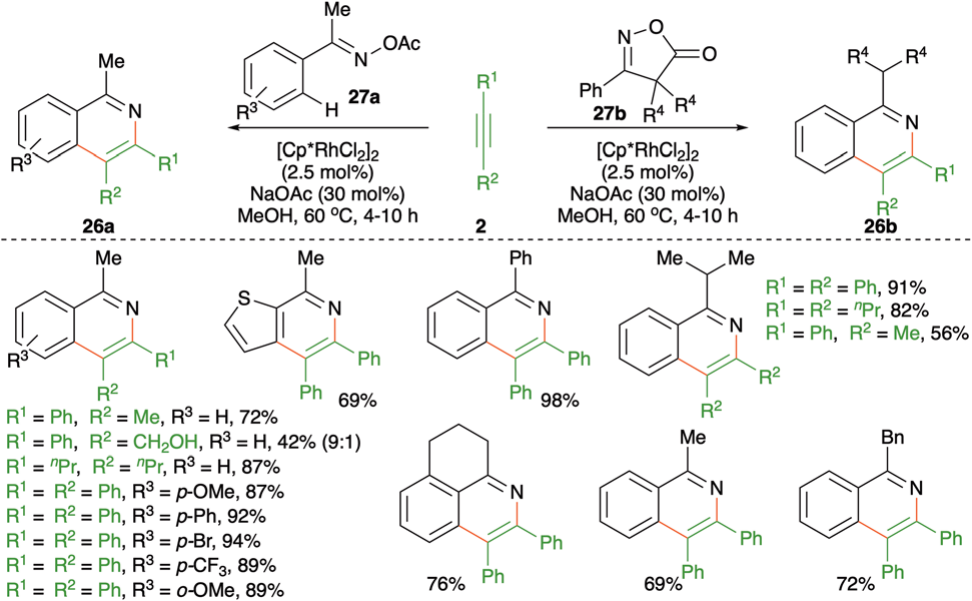
Carboamination of alkynes with imines and oxazoles.

Similar to the previous reports, Chiba and co-workers reported a method describing the synthesis of isoquinolines to form corresponding oximes or isoxazoles (Scheme 15).^26^ The method involved treatment of oximes or isoxazoles 27a,b with alkynes 2 in the presence of 2.5 mol% [Cp*RhCl_2_]_2_ to furnish isoquinolines 26a,b in good yield. The reaction worked well for both aliphatic and aromatic alkynes. However, unsymmetrical alkynes produced a mixture of regio-isomeric products.

**Scheme 16:**
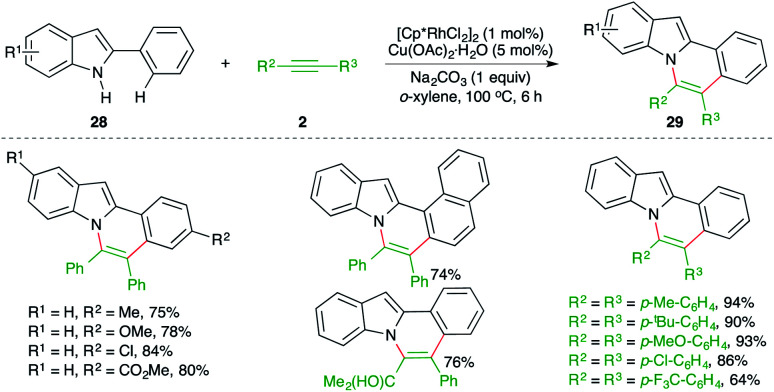
Carboamination of alkynes with 2-aryl indoles.

The intermolecular carboamination strategy has also been applied for the construction of highly promising *N*-fused indole motifs. The reaction of 2-arylindoles 28 with various alkynes 2 in the presence of 1 mol% [Cp*RhCl_2_]_2_ and copper acetate as oxidant furnished the required *N*-fused indoles 29 in good yield (Scheme 16).^27^

**Scheme 17:**
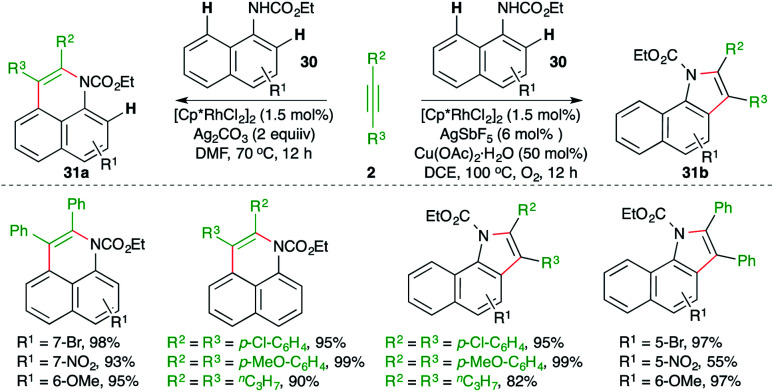
Carboamination of alkynes with naphthyl carbamates.

A diverse Rh-catalyzed approach for the construction of benzoquinolines and benzoindoles from naphthyl carbamates through the engagement of a carboamination strategy was elaborated by Jin and co-workers (Scheme 17).^28^ The method consisted of reaction of naphthyl carbamates 30 with alkynes 2 in the presence of a Rh-catalyst to generate different N-heterocycles 31a,b. The formation of the different products was dictated by the additive. The use of copper additive made neutral rhodium into cationic rhodium, which governed the *ortho* C–H activation and led to the formation of benzoindoles 31b. On the other hand, additives like silver accelerated the *peri* C–H activation to furnish benzoquinolines 31a.

**Scheme 18:**
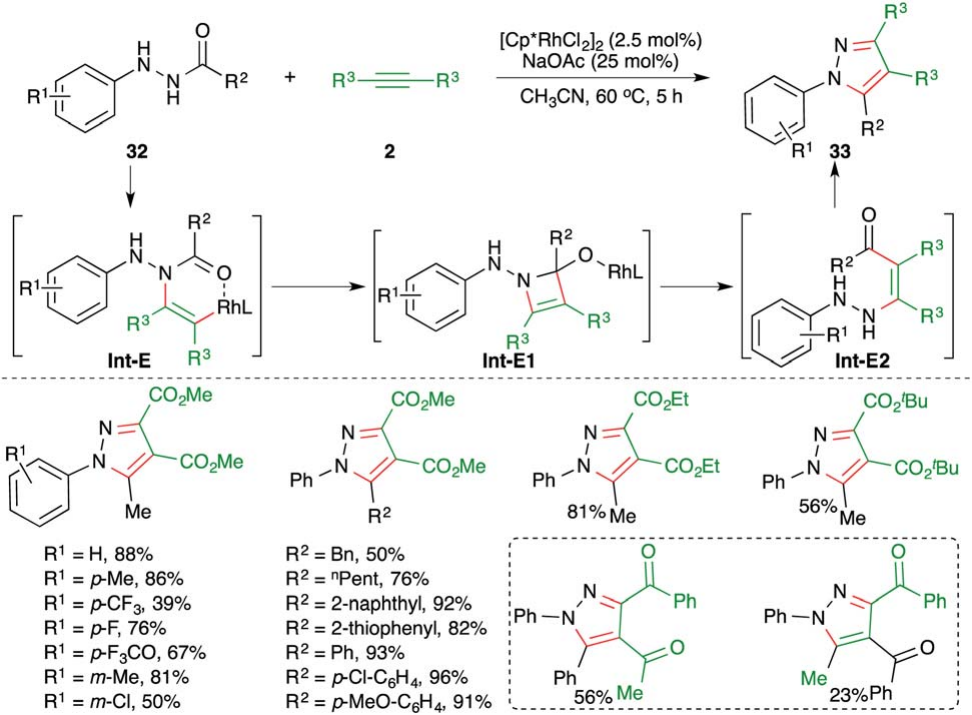
Carboamination of alkynes with hydrazines.

An Rh-catalyzed intermolecular carboamination–cyclization cascade was achieved by Liu and co-workers for the quick synthesis of pyrazole derivatives. The reaction of *N*-acyl hydrazines 32 with electron-deficient alkynes 2 in the presence of 2.5 mol% [Cp*RhCl_2_]_2_ gave the corresponding pyrazole derivatives 33 in good yield (Scheme 18).^29^ Various alkynes were used in the transformation to obtain the corresponding pyrazoles in good yield. However, alkynones gave a mixture of products and the minor one was formed *via* condensation with the keto group of the alkynes. The reaction mechanism involved hydroamination of alkynes through Rh-activation of alkynes to generate rhodacycles Int-E, which upon subsequent intramolecular cyclization led to the formation of Int-E1. The Int-E1 underwent C–N bond cleavage to give the carboamination adducts Int-E2. Finally, intramolecular condensation furnished the desired pyrazoles.

**Scheme 19:**
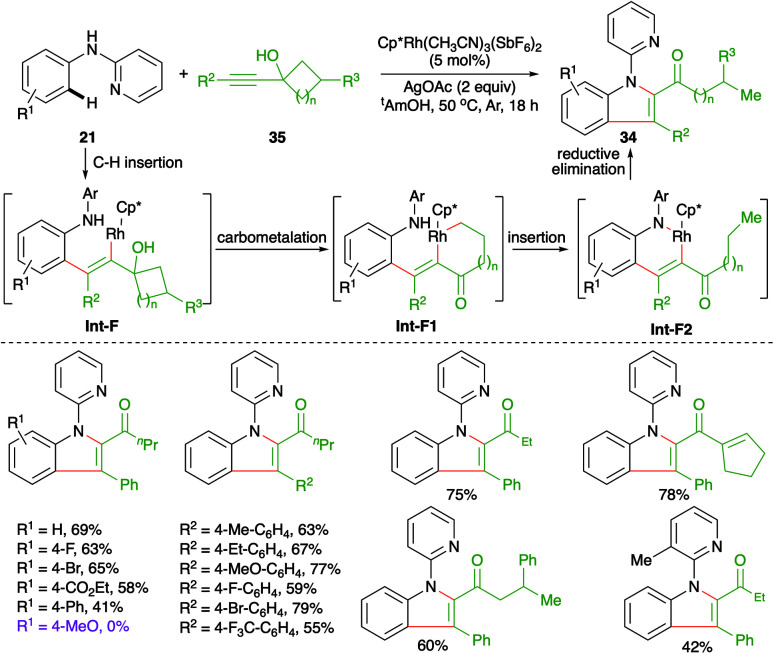
Carboamination of alkynes with anilines.

Carboamination of alkynes has emerged as a powerful tool for the synthesis of various enamines. Further, these types of cascades are being used frequently in the literature to synthesize heteroaromatic entities. In this direction, Zeng *et al.* have reported the synthesis of 2,3-disubstituted indoles 34 from internal alkynols 35 and directing group-tethered aniline derivatives 21 by using an Rh-catalyzed intermolecular carboamination strategy (Scheme 19).^30^

The cascade involved Rh-catalyzed addition at the *ortho* C–H bond in alkynes to generate rhodacycles Int-F1, which upon nucleophilic attack by nitrogen onto rhodium furnished Int-F2. Finally, reductive elimination of rhodium from Int-F2 furnished 2,3-disubstituted indoles 34. In general, it was observed that alkynols with electron-donating substituents on the aryl ring failed to give the desired indole derivatives.

**Scheme 20:**
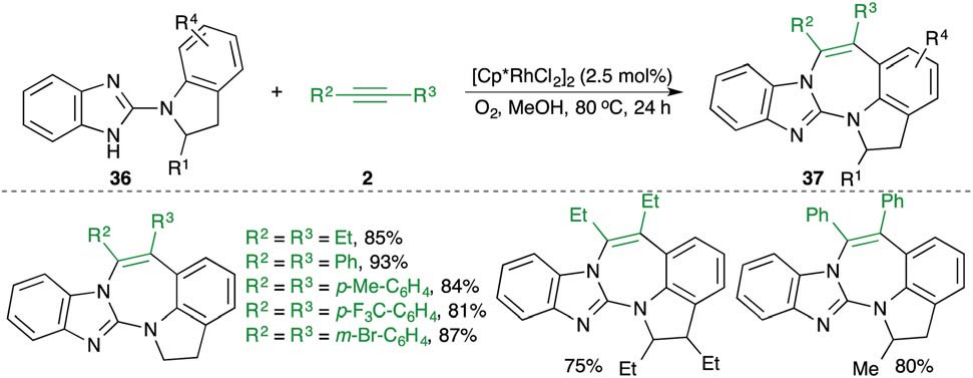
Carboamination of alkynes with arylguanidines.

A Rh-catalyzed carboamination reaction of arylguanidines 36 and alkynes 2 for the quick synthesis of 1,3-benzodiazepines 37 was studied by Saá and co-workers (Scheme 20).^31^ In general, it was observed that use of molecular oxygen as oxidant was accompanied by better yields in comparison with the traditional oxidants like silver. Both aromatic and aliphatic alkynes were used in the reaction.

**Scheme 21:**
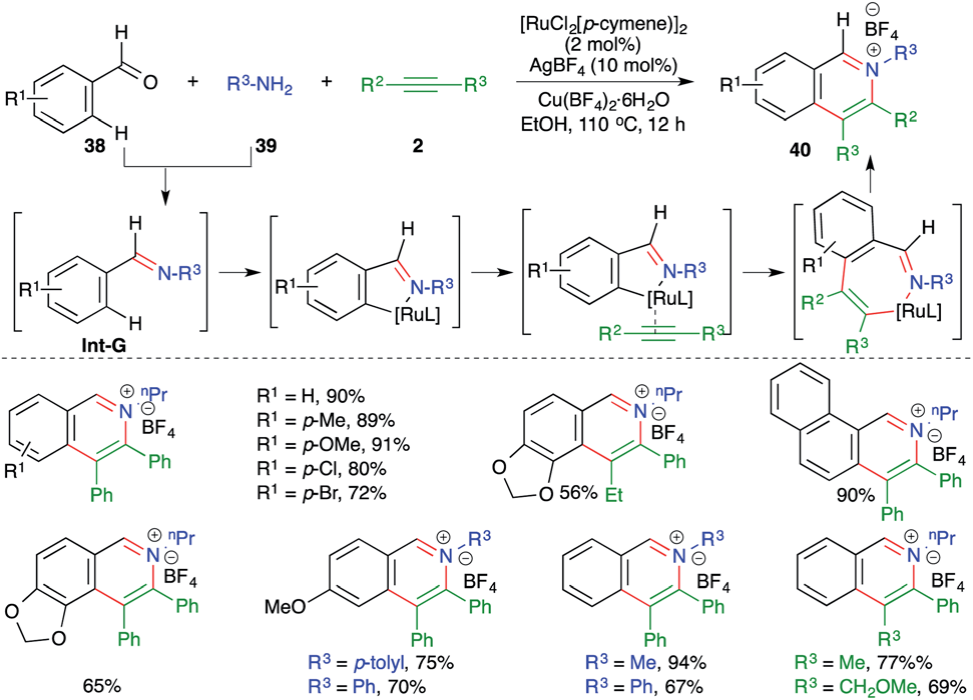
A three-component carboamination approach.

A multicomponent approach using Ru-catalyzed intermolecular carboamination of alkynes was outlined by Cheng and co-workers for the expedient synthesis of isoquinolium salts. The method involved the reaction of aldehydes 38 with amines 39 and alkynes 2 in the presence of 2 mol% [RuCl_2_(*p*-cymene)]_2_ and 10 mol% AgBF_4_ to give the corresponding isoquinolium salts 40 in good yield (Scheme 21).^32^ The reaction mechanism involves condensation of amines 38 with aldehydes 39 to generate imines Int-G, which upon subsequent C–H insertion, carbometalation to alkenes and reductive elimination furnish isoquinolium salts 40.

**Scheme 22:**
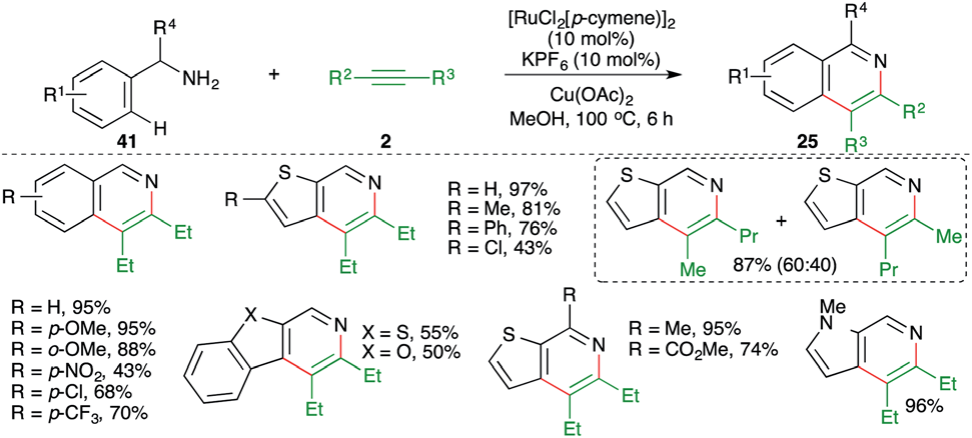
Carboamination of alkynes with benzyl amines.

A directing group-free, Ru-catalyzed carboamination approach was demonstrated by Urriolabeitia and co-workers for the quick synthesis of isoquinolines. The reaction involved treatment of benzyl amine derivatives 41 with alkynes 2 in the presence of 10 mol% [RuCl_2_(*p*-cymene)]_2_ and gave the corresponding isoquinolines 25 in good yield. Further, the method was also applied to the synthesis of benzoisoquinolines, thieno[3,2-*c*]pyridines, and fused heteroaryl[2,3-*c*]pyridines (Scheme 22).^33^

**Scheme 23:**
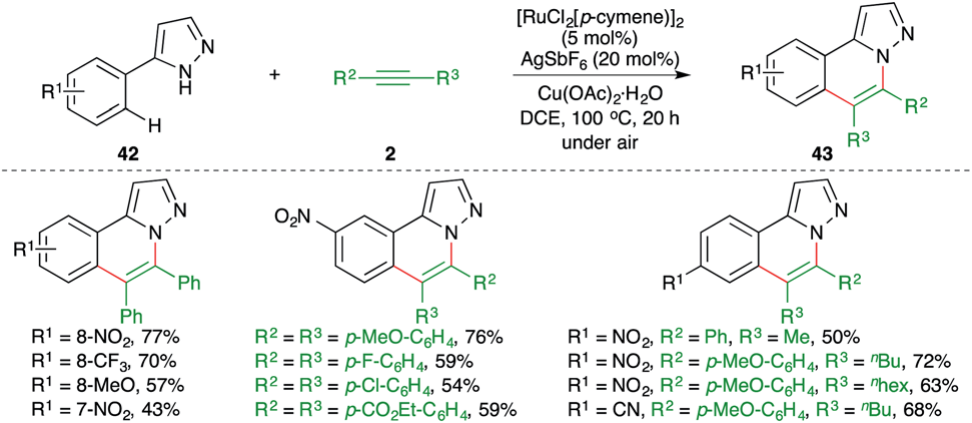
Carboamination of alkynes with pyrazole-tethered arenes.

Ru-catalyzed carboamination reaction of 1*H*-pyrazole-tethered arenes was elaborated by Ackermann and co-workers for the facile synthesis of substituted 1*H*-pyrazole derivatives (Scheme 23).^34^ The method involved treatment of arenes 42 with alkynes 2 in the presence of 5 mol% [RuCl_2_(*p*-cymene)]_2_ to give the desired pyrazoles 43 in good yield. Intriguingly, in the case of unsymmetrical alkynes, the developed method exhibited excellent regioselectivity, leading to the exclusive formation of a single regio-isomer.

**Scheme 24:**
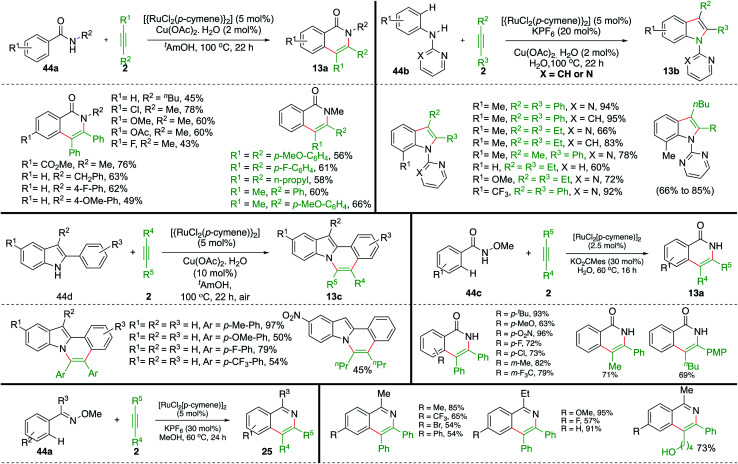
Carboamination of alkynes with aryl oximes and aryl amides.

In addition to the above report, Ackermann and co-workers have also studied extensively this type of Ru-catalyzed intermolecular carboamination of various aryl amides with alkynes for the synthesis of an avenue of N-heterocycles. The aryl amides 44a upon reaction with alkynes 2 in the presence of 5 mol% Ru-catalyst gave the corresponding isoquinolones 13a in good yield. Various isoquinolones were synthesized successfully using both symmetrical as well as unsymmetrical alkynes. It is pertinent to mention that, in the case of unsymmetrical alkynes, excellent regioselectivity was observed, leading to the formation of a single regio-isomer. On similar lines, they have also reported synthesis of variously substituted indoles 13b using the Ru-catalyzed intermolecular carboamination of alkynes 2 with directing-group-tethered anilines 13b (Scheme 24). Further, they extended their strategy for the synthesis of isoquinolones 13a and N-fused indoles 13c from the corresponding hydroxamic acids 44c and indole-tethered arenes 44d.

Variously substituted N-heterocycles were successfully synthesized in good yield and with excellent regioselectivity using both symmetrical as well as unsymmetrical alkynes. In this context, Ackermann's group and Jeganmohan *et al.* studied synthesis of isoquinolines 25 from corresponding oximes 44a using Ru-catalysis.^35–39^

**Scheme 25:**
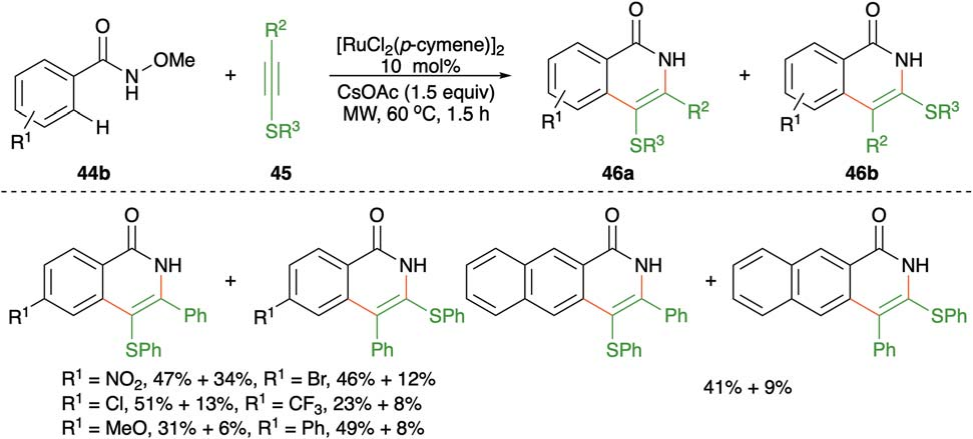
Carboamination of alkynes with aryl oximes.

On similar lines, Yao and co-workers have used aryl/alkyl(phenylethynyl)sulfanes instead of simple alkynes for the synthesis of *S*-tethered isoquinolones. The approach involved the reaction of *N*-methoxy aryl amides 44b with aryl/alkyl(phenylethynyl)sulfanes 45 in the presence of 10 mol% [RuCl_2_(*p*-cymene)]_2_ to afford a mixture of *S*-tethered isoquinolones 46b in good yield (Scheme 25).^40^ Although this was the first report on the reaction of these types of alkynes, they were unable to produce exclusively a single regio-isomer.

**Scheme 26:**
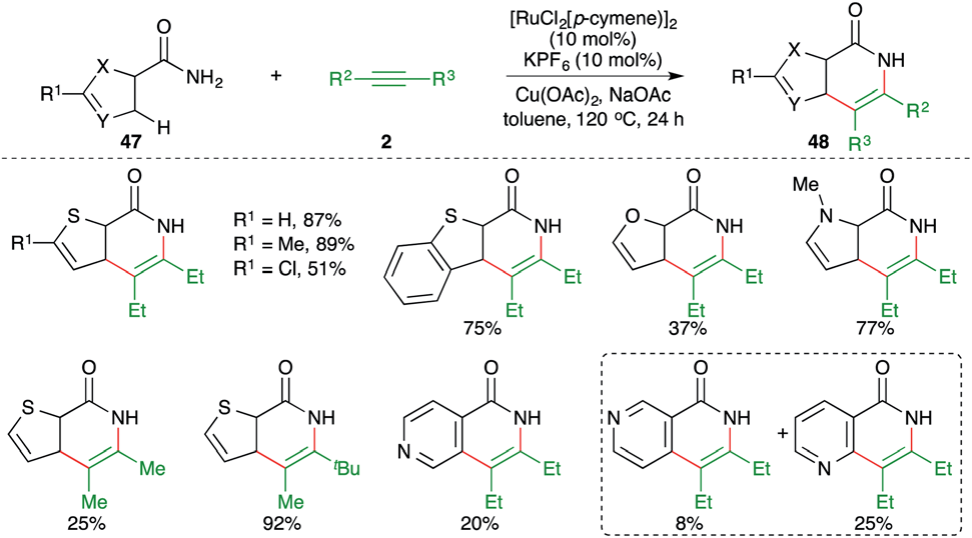
Carboamination of alkynes with aryl amides.

Reactions involving C–H activation of free amides are difficult as mostly the C–H activation requires directing group assistance for the C–H insertion of metal. However, methods describing directing group-free C–H activations have been documented in the literature. In this direction, Urriolabeitia and co-workers have reported a carboamination reaction of directing group-less heteroarene-tethered amides 47 with alkynes 2 in the presence of a Ru-catalyst to give the corresponding heteroarene-fused quinolones 48 in good yield (Scheme 26).^41^ The reaction worked well for 5-membered heteroarene-fused (thiophene) amides, whereas in the case of 6-membered heteroarenes, poor yields of the corresponding quinolones were obtained. Further, in cases where there were two potential sites of C–H insertion, a mixture of products was obtained. Interestingly, in the case of unsymmetrical alkynes, only one product was obtained.

**Scheme 27:**
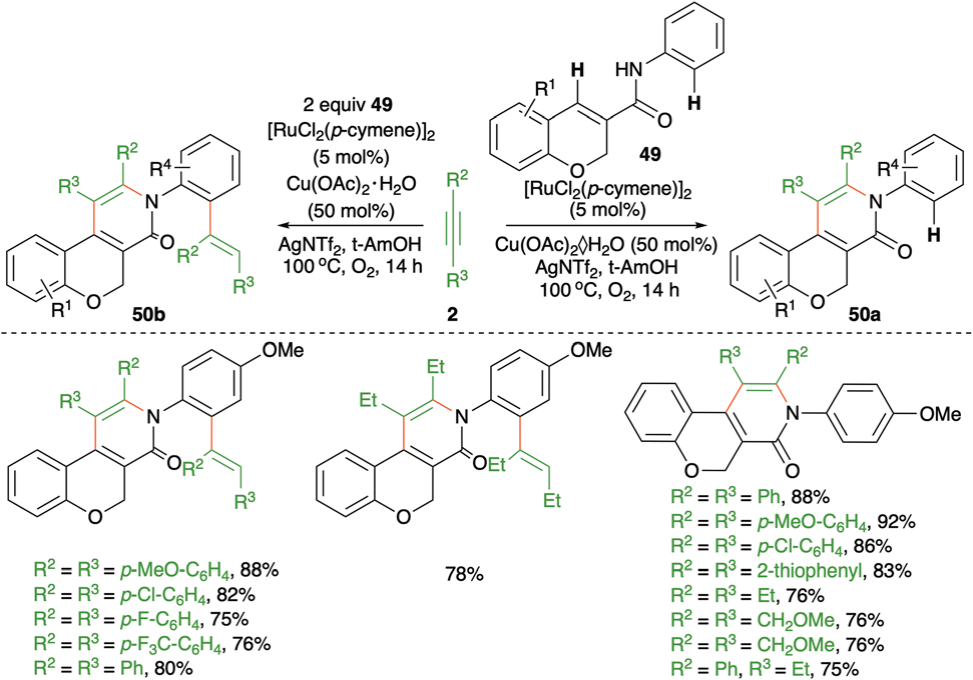
Carboamination of alkynes with aryl amides.

Regioselective C–H functionalization of chromene-3-carboxamides was described by Swamy and co-workers using a Ru-catalyzed intermolecular carboamination reaction (Scheme 27).^42^ The method involved the reaction of chromene-3-carboxamides 49 with alkynes 2 in the presence of 5 mol% [RuCl_2_(*p*-cymene)]_2_ to furnish pyridinones 50a in good yield *via* the selective functionalization of the benzylic C–H bond. However, use of excess alkynes led to the formation of vinyl-substitute pyridinones 50b through double C–H activation. Interestingly, in the case of unsymmetrical alkynes, *i.e.*, aliphatic–aromatic alkynes, a single regio-isomer was formed. In contrast, alkynes having different aryl substituents led to a mixture of regio-isomers.

**Scheme 28:**
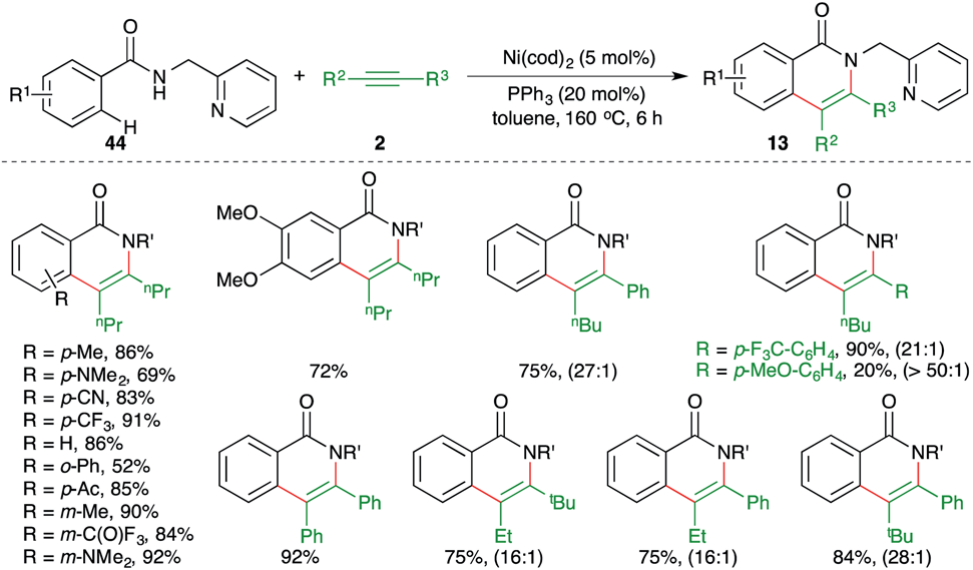
Carboamination of alkynes with aryl amides.

Chatani and co-workers have reported a chelation-assisted strategy for the synthesis of quinolones by using Ni-catalyzed carboamination (Scheme 28).^43^

The method involved treatment of directing group *i.e.*, pyridine-tethered benzamides 44 with alkynes 2 in the presence of 5 mol% Ni(cod)_2_ to form the corresponding isoquinolones 13 in good yield. The reaction has a very broad scope with excellent functional group tolerance. Further, both aliphatic as well as aromatic internal alkynes were used in the transformation. Intriguingly in the case of unsymmetrical alkynes, excellent regioselectivity was observed; the ratios are given in parentheses in Scheme 28.

**Scheme 29:**
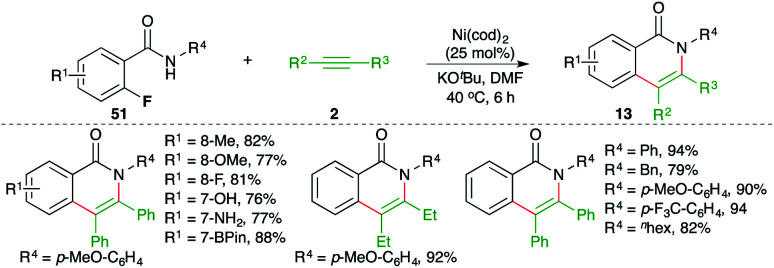
Carboamination of alkynes with *o*-fluoro aryl amides.

Ni-catalyzed carboamination reaction involving tandem C–F and N–H bond activation was elaborated by Chatani and co-workers for the facile synthesis of isoquinolones (Scheme 29).^44^ The reaction of *o*-fluoro aryl amides 51 with alkynes 2 in the presence of Ni(cod)_2_ gave the corresponding isoquinolones 25 in good yield. Although the reaction had a very broad scope, it was limited to internal alkynes only. Further, in the case of unsymmetrical alkynes, a mixture of regio-isomers was obtained.

**Scheme 30:**
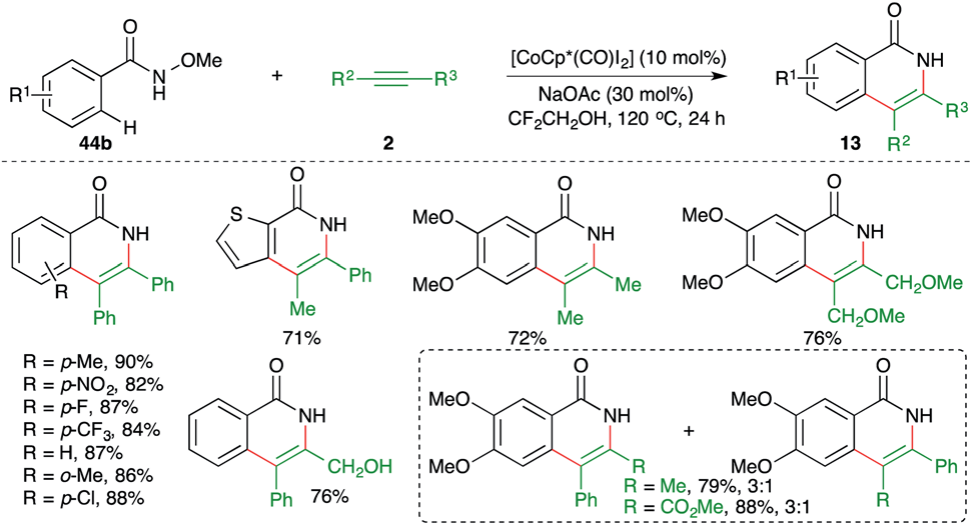
Carboamination of alkynes with aryl amides.

Jeganmohan *et al.* studied the feasibility of similar carboamination approaches with different metals and they found that other than Rh-metal, cobalt could drive the cascade. The reaction of *N*-aryl oximes 44b with alkynes 2 in the presence of [CoCp*(CO)I_2_] gave the corresponding isoquinolones 13 in good yield (Scheme 30).^45^

**Scheme 31:**
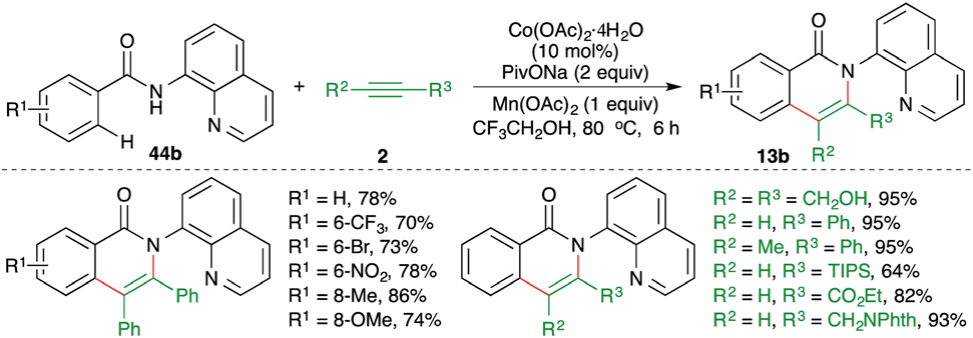
Carboamination of alkynes with directing group-tethered aryl amides.

On similar lines, a Co-catalyzed directing group-assisted intermolecular carboamination of alkynes was studied by Daugulis and co-workers for the synthesis of isoquinolones (Scheme 31).^46^ The reaction of 8-amino quinoline-tethered aryl amides 44b with alkynes 2 in presence of 20 mol% Co(OAc)_2_ gave the corresponding isoquinolones 13b in good yield. Both internal, as well as terminal, alkynes were employed in the reaction to furnish the desired product in good yield.

**Scheme 32:**
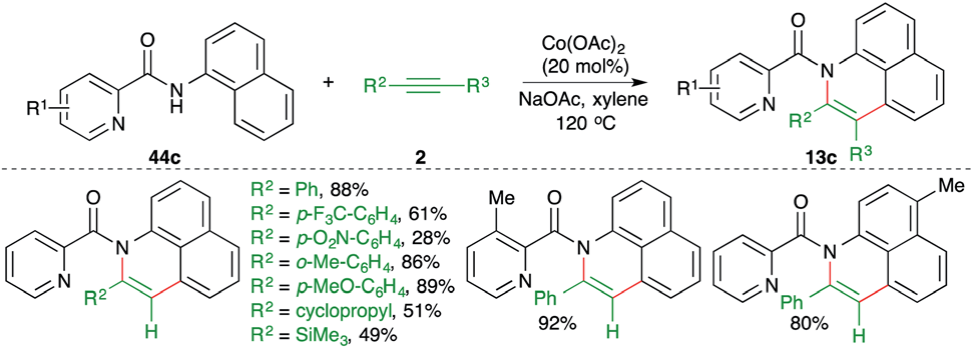
Carboamination of alkynes with directing group-tethered aryl amides.

A cobalt-catalyzed directing group-assisted carboamination strategy was described by Yang and co-workers for the construction of benzoquinolines (Scheme 32).^47^ The reaction of picolinamides 44c with alkynes 2 in the presence of 20 mol% cobalt acetate delivered the corresponding benzoquinolines 13c in good yield. Both internal alkynes and terminal alkynes were used in the transformation. Aryl alkynes with electron-withdrawing groups on the aryl ring gave a lesser yield.

**Scheme 33:**
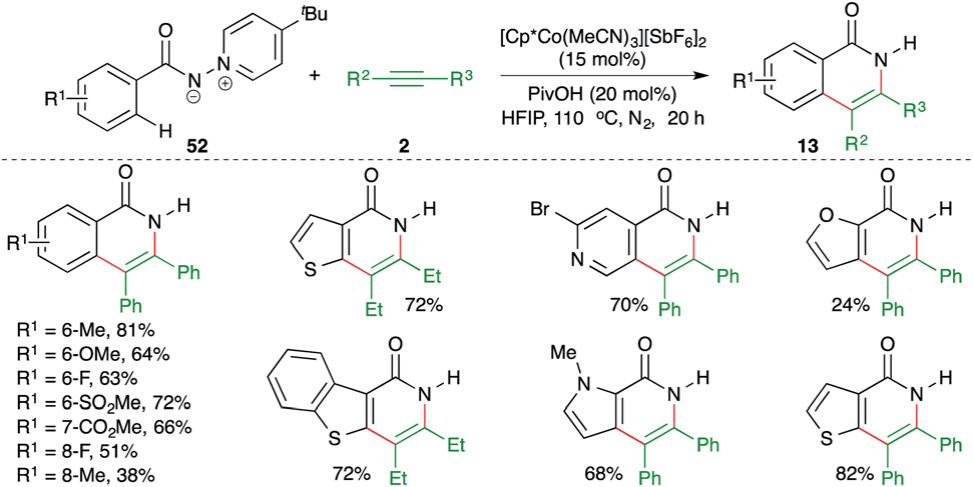
Carboamination of alkynes with ylide-tethered aryl amides.

Ylide, as a directing group for the assisted C–H activation is not so common in the literature despite the fact that it is of equal capability in comparison with the bidentate auxiliaries. In this context, Daugulis and co-workers have reported an *N*-iminopyridinium ylide-directed, cobalt-catalyzed carboamination reaction of aryl amides 52 with alkynes 2 for the synthesis of isoquinolones 13 in good yield (Scheme 33).^48^ Interestingly, the directing group was removed in the same pot.

**Scheme 34:**
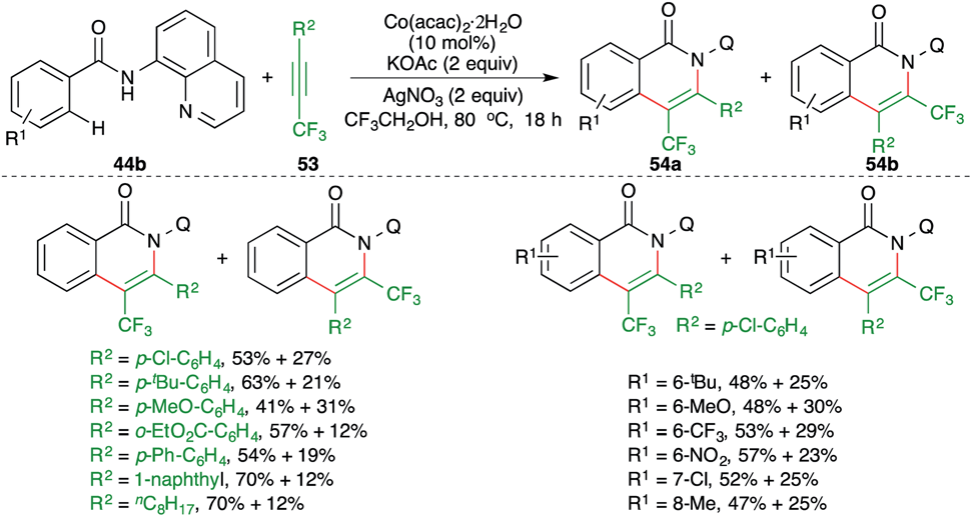
Carboamination of fluoroalkylated-alkynes with directing group-tethered aryl amides.

Fluoroalkylated alkynes have also been used in the carboamination process involving C–H activation. Konno and co-workers have studied a cobalt-catalyzed carboamination reaction of directing group-tethered aryl amides 44b with fluoroalkylated alkynes 53 to give the corresponding isoquinolones 54a,b in good yield (Scheme 34).^49^ Although the method gave rapid access to the fluorinated isoquinolones, a mixture of regio-isomers was obtained in all cases. Furthermore, the reaction only worked with 8-quinolinyl-masked amides.

#### 2.1.3 Carboamination triggered by cycloaddition

**Scheme 35:**
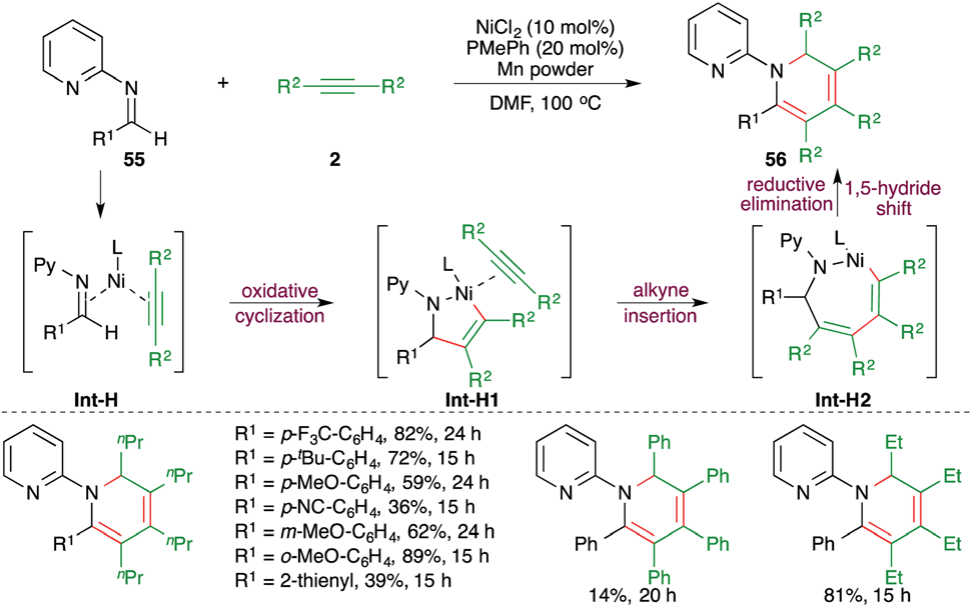
Ni-catalyzed carboamination of alkynes with imines for the synthesis of dihydropyridines.

Ni-catalyzed [2 + 2 + 2] cycloaddition or iterative carboamination of imines 55 with alkynes 2 has been reported by Yoshikai and co-workers for the facile synthesis of substituted dihydropyridiene derivatives 56 in good yield (Scheme 35).^50^ In general, it was observed that aliphatic alkynes offered a better yield of the desired product in comparison with aromatic alkynes. The reaction mechanism involves oxidative cyclization to form Int-H1, which upon alkyne insertion leads to the formation of Int-H2. Finally, a reductive elimination–1,5-hydride shift cascade furnishes 56.

**Scheme 36:**
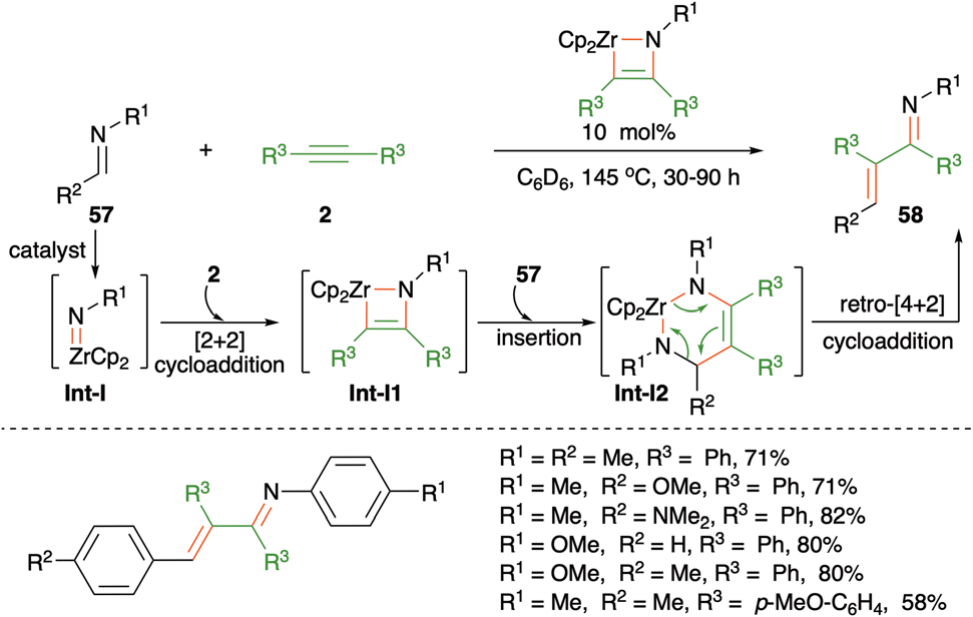
Synthesis of azadienes using the Zr-catalyzed carboamination of alkynes.

The use of imidozirconium complexes as catalyst has provided a vital solution to many organometallic problems such as hydroamination of alkynes or of allenes. In this direction, Bergmann and co-workers have reported imidozirconium complex-catalyzed carboamination of alkynes 2 with imines 57 for the generation of α,β-unsaturated imines, *i.e.* enimines, 58 in good yield (Scheme 36).^51^ The formation of product can be explained through Int-I-I2*via* cascade [2 + 2] cycloaddition, insertion/carbozirconation, and retro [4 + 2] cycloaddition process, respectively.

**Scheme 37:**
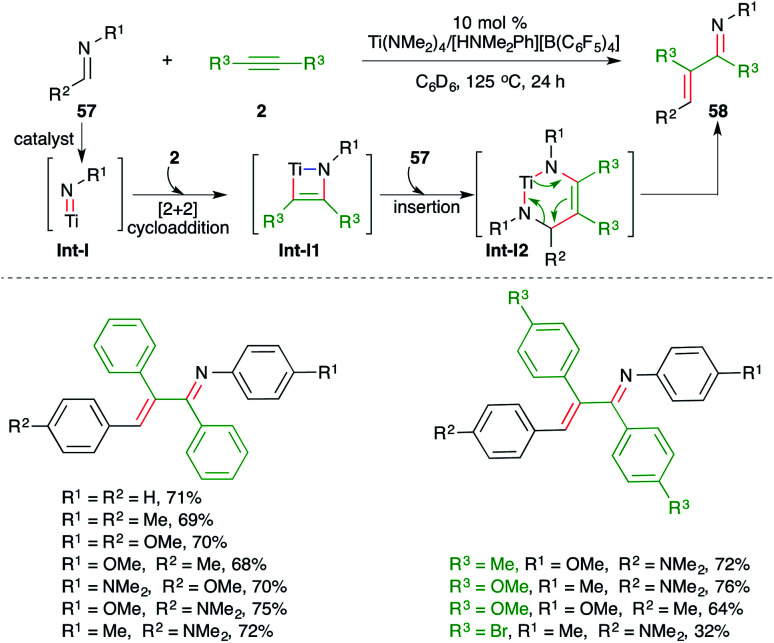
Synthesis of azadienes using the Ti-catalyzed carboamination of alkynes.

On similar lines, a catalytic carboamination process of alkynes using a Ti(NMe_2_)_4_/[HNMe_2_Ph][B(C_6_F_5_)_4_] combined catalytic system was studied by Mindiola and co-workers for the facile synthesis of enimines 58 from corresponding alkynes 2 and imines 57 (Scheme 37).^52^ Although the reaction gave direct access to various enimines, it was limited to symmetrical alkynes, aromatic alkynes, and imines from aromatic aldehydes.

**Scheme 38:**
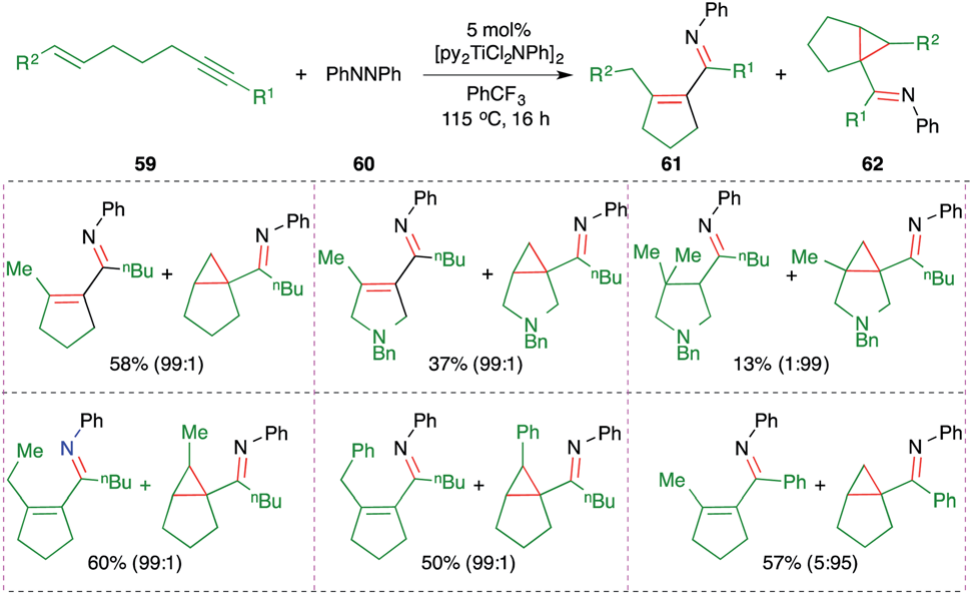
Synthesis of cyclic azadienes using the Ti-catalyzed carboamination of alkynes.

A titanium-catalyzed intermolecular carboamination reaction of alkynes 59 with hydrazines 60 for the synthesis of enimines 61 or imine-tethered cyclopropanes 62 was studied by Tonks and co-workers (Scheme 38).^53^ Treatment of alkene-tethered alkynes with diazine in the presence of 5 mol% [py_2_TiCl_2_NPh]_2_ gave the corresponding enamine- or imine-tethered cyclopropanes in good yield.

**Scheme 39:**
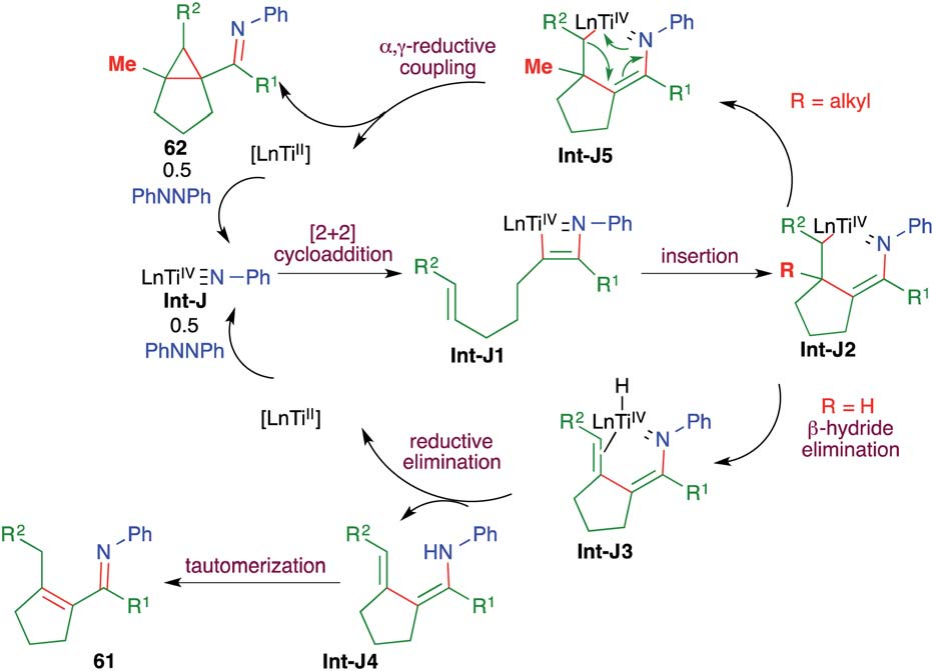
Reaction mechanism for obtaining 61 and 62.

The reaction mechanism involves the initial formation of Int-J by the combination of hydrazine 60 and [py_2_TiCl_2_NPh]_2._ The Int-J upon [2 + 2] cycloaddition reaction with alkynes 59 generates Int-J1. The Int-J1 gives a Ti-incorporated 6-membered cyclic transition state Int-J2*via* intramolecular insertion. Interestingly, if R = H in Int-J2, then β-hydride elimination takes place to give Int-J3. Finally, reductive elimination of titanium from Int-J3 gives enamines Int-J4, which upon subsequent tautomerization lead to the formation of enimines 61. On the other hand, if R = alkyl in Int-J2, then instead of β-hydride elimination α,γ-reductive coupling takes place to give cyclopropane 62*via*Int-J5 (Scheme 39).

**Scheme 40:**
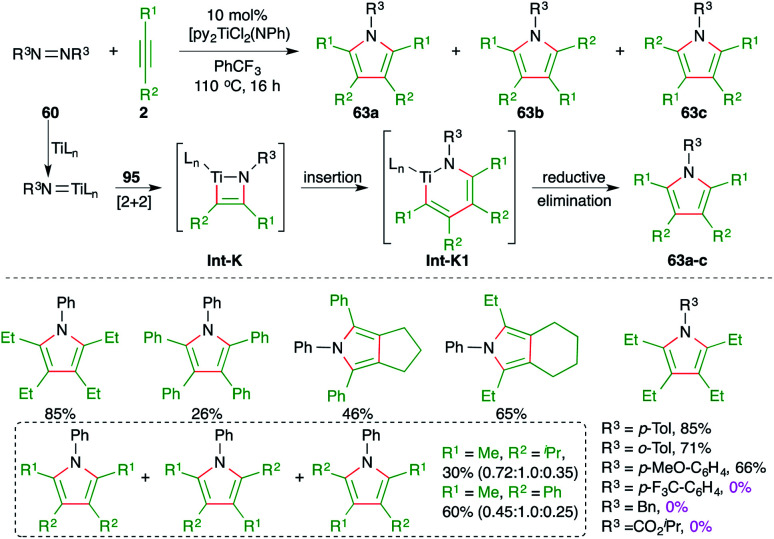
Synthesis of pyrroles using the Ti-catalyzed carboamination of alkynes.

Furthermore, Tonks and co-workers have also developed a novel three-component oxidative C–N bond formation reaction with titanium catalyst. The reaction involved [2 + 2 + 1] cyclization reaction of various alkynes 2 and diazenes 60 to furnish the respective polysubstituted pyrroles 63a–c in good yield (Scheme 40).^54^ The reaction was proposed to proceed *via* a Ti(ii)/Ti(iv) redox cycle where an azatitanacyclobutene intermediate Int-K is formed from the [2 + 2] addition of one equivalent of alkynes and titanium imido complex (py)_3_TiCl_2_(NR). Another equivalent of alkynes undergoes insertion into the Int-K, forming an azatitanacyclohexadiene Int-K1, followed by reductive elimination to give the pyrroles 63a–c. In general, the reaction worked well with less hindered alkynes. Further, in the case of unsymmetrical alkynes, a mixture of regio-isomers was obtained. It was also observed that aryl diazines with electron-withdrawing substituents could not deliver the required product.

#### 2.1.4 Carboamination of benzynes

Benzynes are very synthetically useful intermediates and in a true sense, they have shown the capacity of difunctionalization of carbon–carbon multiple bonds. Although they are very unstable, their *in situ* generation and cascade reactivity have provided a new route to a suite of really interesting building blocks. In this domain, carboamination of benzyne has also been reported.

**Scheme 41:**
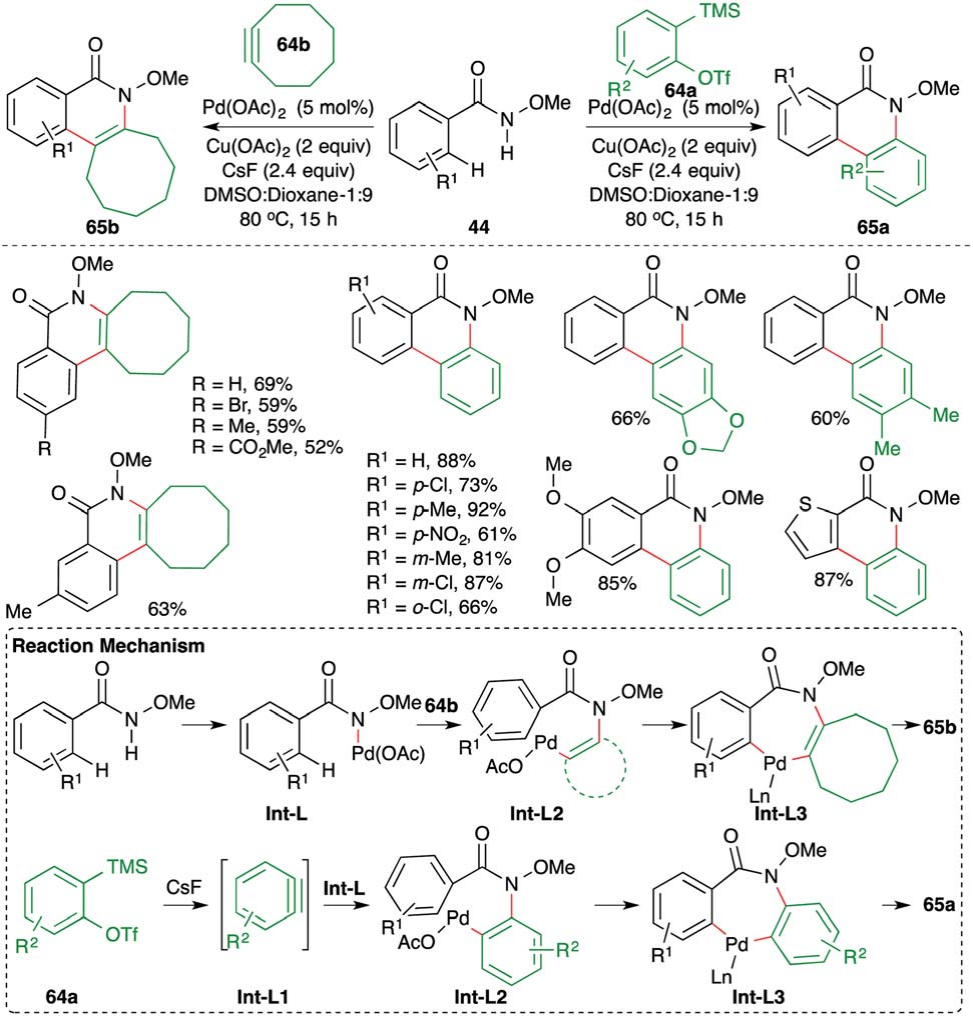
Carboamination of benzynes with amides.

A Pd-catalyzed intermolecular carboamination of arynes strategy was developed by Xu and co-workers for rapid access to highly functionalized N-heterocycles (Scheme 41).^55^ The method involved the reaction of aryl-tethered *N*-methoxy amides 44 with 2-(trimethylsilyl)aryl triflates 64a and strained alkynes 64b in the presence of 5 mol% Pd(OAc)_2_ to give the corresponding N-heterocycles 65a,b, respectively, in good yield. Both electron-rich and electron-poor amides participated equally in the reaction, leading to the formation of the desired N-heterocycles. The reaction proceeds through the formation of Int-L*via* N–H insertion, which on amino-palladation with alkynes 64b or benzynes Int-L1 (generated *in situ* from 64a) gives Int-L2. Int-L2 upon C–H activation generates palladacycles Int-L3. Finally, reductive elimination of palladium from Int-L3 leads to the formation of 65a,b.

**Scheme 42:**
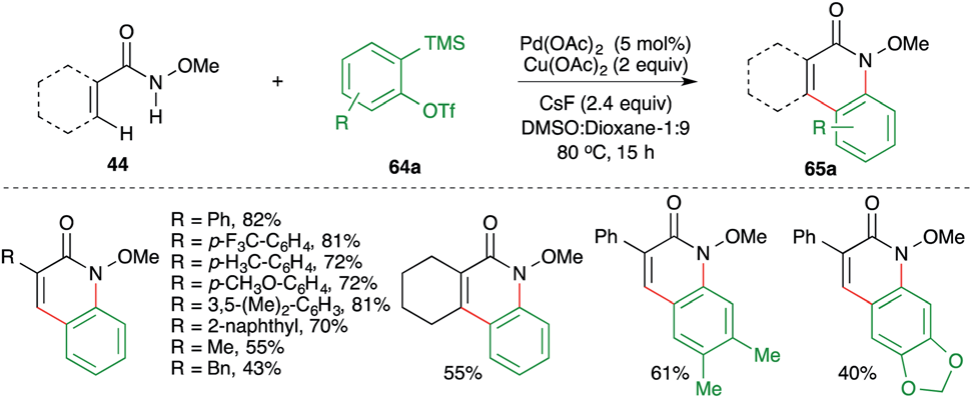
Synthesis of *N*-methoxy isoquinolones using the carboamination of benzynes.

On similar lines, Tonks and co-workers have also reported such a cascade for the synthesis of isoquinolones starting from a comparatively difficult precursor for C–H activation, *i.e.* non-tethered *N*-methoxy amides (Scheme 42).^56^

The method involved treatment of *N*-methoxy amides 44 with 2-(trimethylsilyl)aryl triflates 64a in the presence of CsF and Pd(OAc)_2_ to give isoquinolones 65a. The method has a very good scope along with good functional group tolerance.

**Scheme 43:**
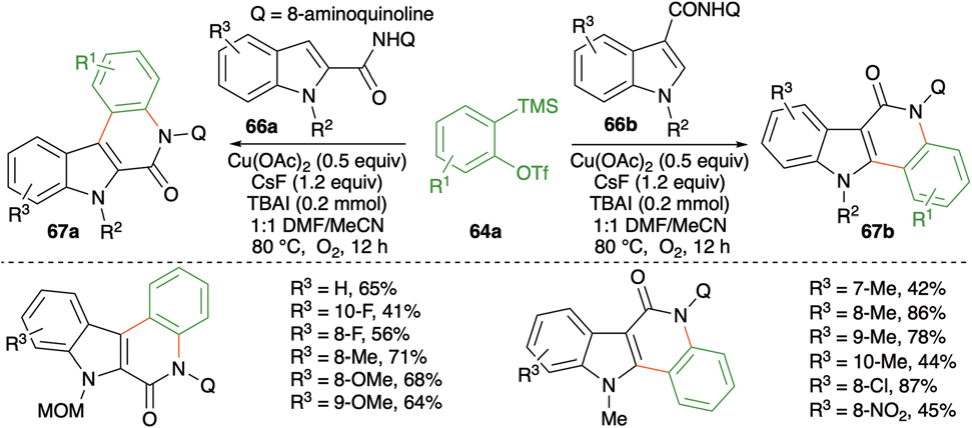
Synthesis of indole-fused isoquinolones using the carboamination of benzynes.

Similarly, instead of aryl amides, the reactivity of indole-tethered amides in this direction was explored by Zhang and co-workers. Reaction of indolyl amides 66a,b with aryne precursor 64a in the presence of Cu(OAc)_2_ (0.5 equiv.), CsF (0.24 mmol), and TBAI (0.2 mmol) furnished the corresponding indolo[3,2-*c*]quinolones 67a and indolo[2,3-*c*]quinolones 67b, respectively (Scheme 43).^57^ In general, it was observed that *N*-alkyl indoles, *N*-MOM, were suitable partners for coupling, whereas *N*-Cbz-protected indoles failed to give the desired product. Further, the yield was found to be dependent more upon steric factors than electronic factors as 5-substituted indoles gave a lower yield of the requisite adducts.

**Scheme 44:**
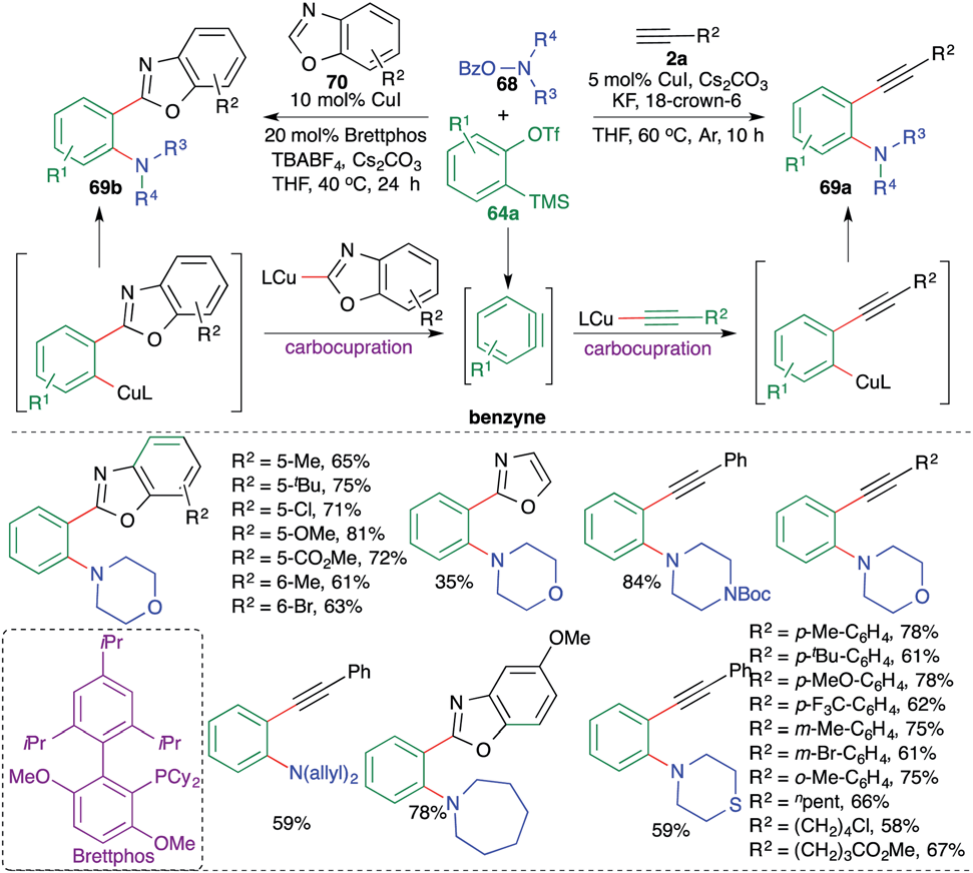
Synthesis of *o*-alkynyl anilines using the carboamination of benzynes.

A copper-catalyzed carboamination of *in situ*-generated benzyne was studied by Xiao and co-workers for the facile synthesis of substituted aniline and *o*-benzoxazolyl aniline derivatives (Scheme 44).^58^

The reaction of various 2-(trimethylsilyl)phenyl trifluoromethanesulfonates 64a with terminal alkynes 2a and *o*-benzoylhydroxylamines 68 in the presence of 5 mol% CuI and KF resulted in the formation of *o*-alkynyl anilines 69a in good yield. The reaction exhibited excellent functional group tolerance and variously substituted anilines were prepared. Aromatic and aliphatic alkynes participated equally well to form the corresponding *o*-alkynyl anilines in good yield. Further, the method was successfully extended towards the replacement of terminal alkynes 2a with benzoxazoles 70 for the synthesis of the corresponding *o*-benzoxazolyl aniline derivatives 69b. However, in this case the reaction conditions had to be adjusted to get the desired products: 20 mol% of Brettphos was used as additive and TBABF_4_ was used instead of KF. The reaction mechanism involved the initial formation of benzyne followed by carboamination reaction.

**Scheme 45:**
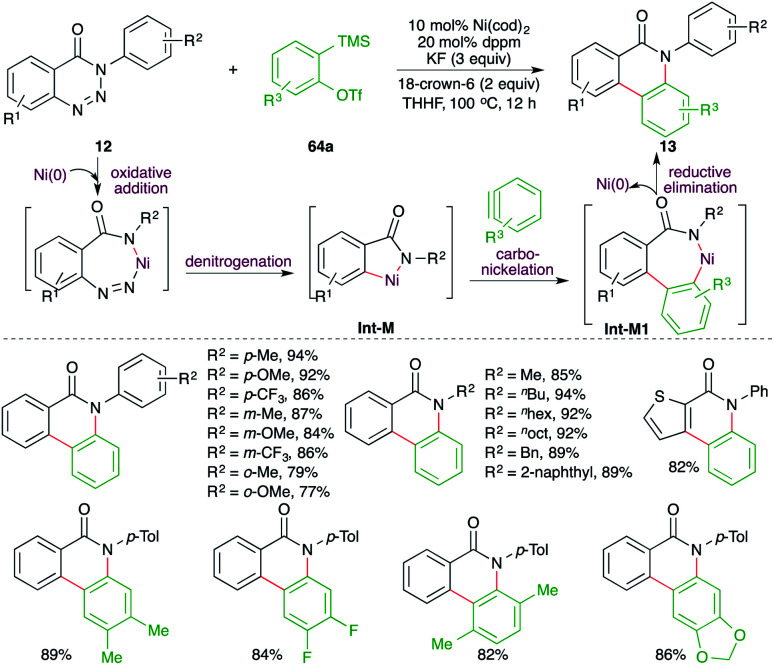
Denitrogenative carboamination cascade of benzynes.

A Ni-catalyzed denitrogenative cascade annulation method for the synthesis of phenanthridinone scaffolds was demonstrated by Cheng and co-workers (Scheme 45).^59^ The report involved treatment of benzotriazin-4-(3*H*)-ones 12 with 2-(trimethylsilyl)aryl triflates 64a in the presence of 10 mol% Ni(cod)_2_, 20 mol% dppm, and 3 equiv. of KF as the fluoride source to generate the corresponding phenanthridinone scaffolds 13 in good yield. The reaction involves fluoride-promoted formation of benzynes, which upon carbo-nickelation reaction with Int-M furnish nickelacycles Int-M1. The Int-M1 follows a reductive elimination path to give 13. The reaction has a very broad substrate scope indeed.

## 3. Intramolecular carboamination

**Scheme 46:**
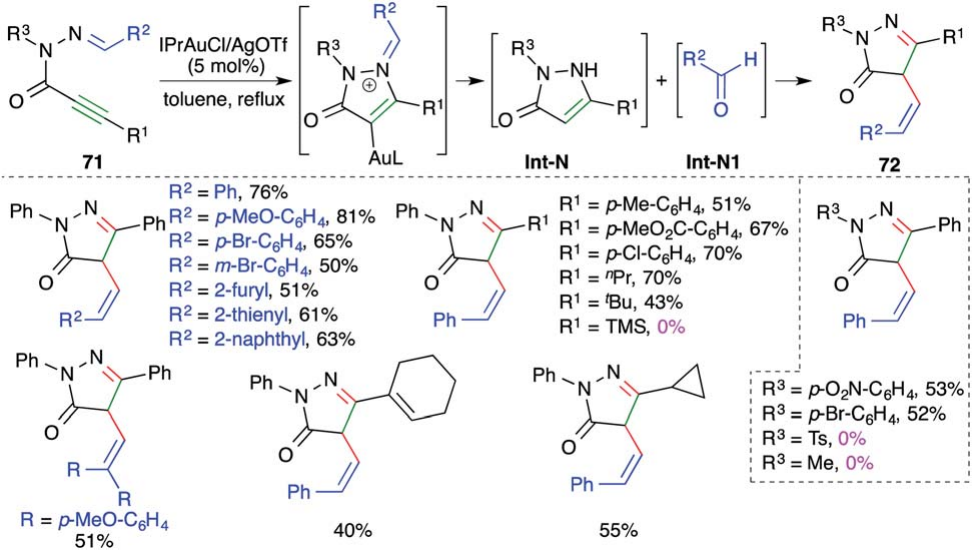
Synthesis of pyrazolidin-3-ones using intramolecular carboamination.

*N*-Propioloyl hydrazones were found to be suitable precursors for the intramolecular carboamination of alkynes, as described by Zhan and co-workers, for the quick synthesis of 4-arylidene pyrazolones. The reaction of alkynones 71 with 5 mol% IPrAuCl and AgOTf delivered the corresponding cyclic derivatives 72 in good yield *via* tandem amination and [1,3]-shift (Scheme 46).^60^ The reaction worked well with aliphatic as well as aromatic alkynes, except TMS-alkynes. Further, in general it was observed that tosyl/aliphatic amides (R^3^ = TMS, alkyl) did not furnish the required products. The formation of the product is explained as follows. Gold catalyzes the intramolecular amination followed by hydrolysis to give enamines Int-N and aldehydes Int-N1. Finally, Int-N and Int-N1 condense together to give 72.

**Scheme 47:**
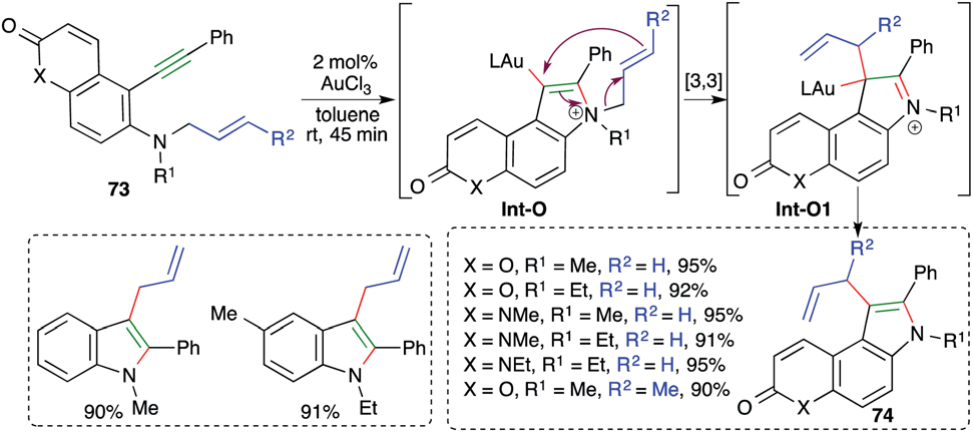
Synthesis of 3-allylated indoles using intramolecular carboamination.

On similar lines, a gold-catalyzed intramolecular carboamination of alkynes was designed by Majumdar and co-workers for the synthesis of functionalized indoles 73 from corresponding *o*-alkynyl-*N*-allyl anilines 74 (Scheme 47).^61^ Interestingly, it was also applied for the synthesis of indoles. However, the reaction had a limited scope for alkynes. The proposed reaction mechanism involves gold-catalyzed intramolecular amination to give iminium ions Int-O, which upon further [3,3]-sigmatropic shift give Int-O1. Finally, deauration furnishes 74.

A gold-catalyzed carboamination cascade was elaborated by Ohno and co-workers for the synthesis of complex N-heterocycles.

**Scheme 48:**
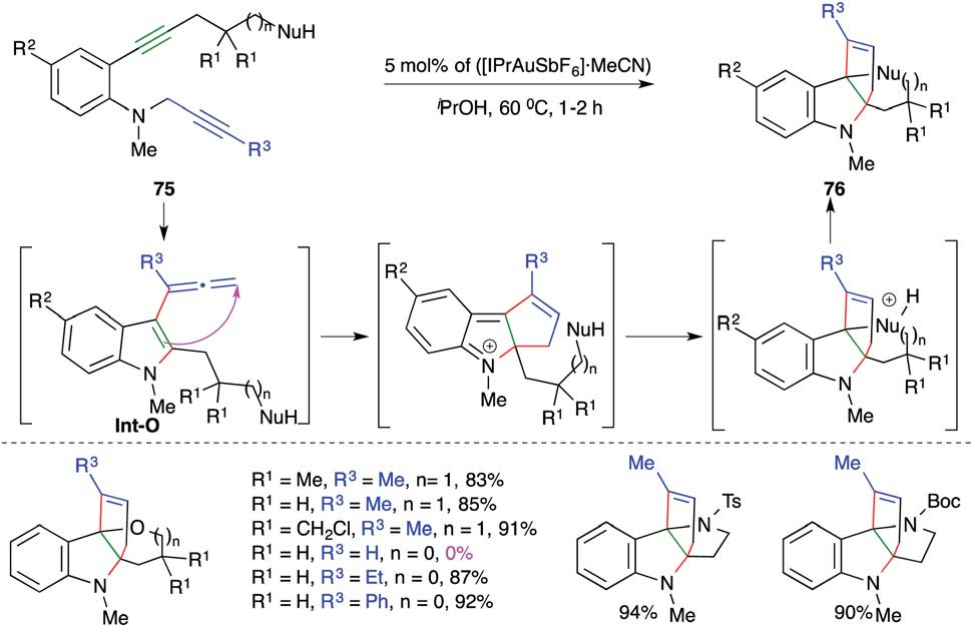
A carboamination–Friedel-Crafts alkylation–intramolecular cyclization cascade.

The cascade involved the reaction of *N*-propargyl *o*-alkynyl anilines 75 with 5 mol% ([IPrAuSbF_6_]·MeCN) to give the corresponding N-heterocycles 76 in excellent yield (Scheme 48).^62^ The carboamination cascade involved intramolecular carboamination to generate allene Int-O (as labelled in Scheme 48), which upon subsequent intramolecular cyclization afforded the desired adducts. The reaction had a broad scope. However, in the case of terminal alkynes, the reaction did not work.

**Scheme 49:**
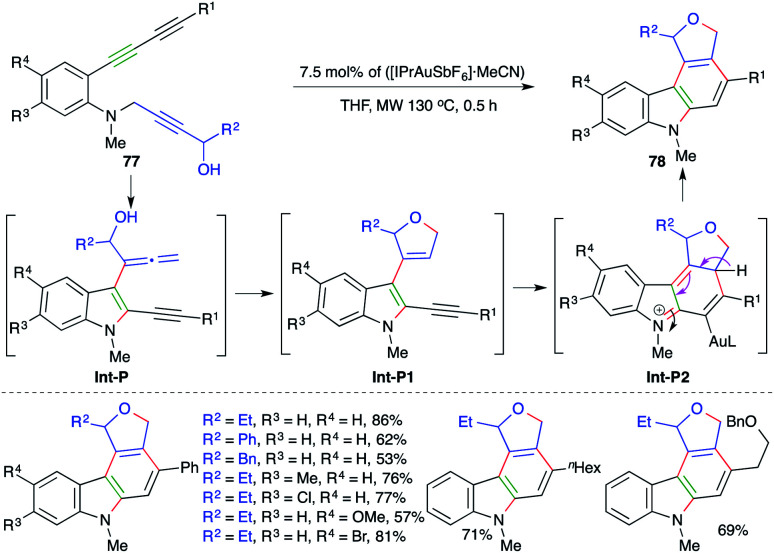
A carboamination–hydroalkoxylation–intramolecular cyclization cascade.

Similar to the above report, Ohno and co-workers have also extended their strategy to a differently designed substrate 77 for the expedient synthesis of cyclic ether/amine-fused carbazoles 78 (Scheme 49).^63^ Various carbazoles were synthesized in good yield. Both internal and terminal alkynes (R^1^ = aryl/alkyl) were employed in the reaction. However, the corresponding terminal alkynes were not used in the transformation. The formation of the product occurs through initial amination and [3,3]-shift to give allenes Int-P. The Int-P upon 5-*endo-trig* cyclization gives Int-P1. Finally, 6-π electrocyclic ring closure of Int-P1 gives Int-P2, which after aromatization affords 78.

**Scheme 50:**
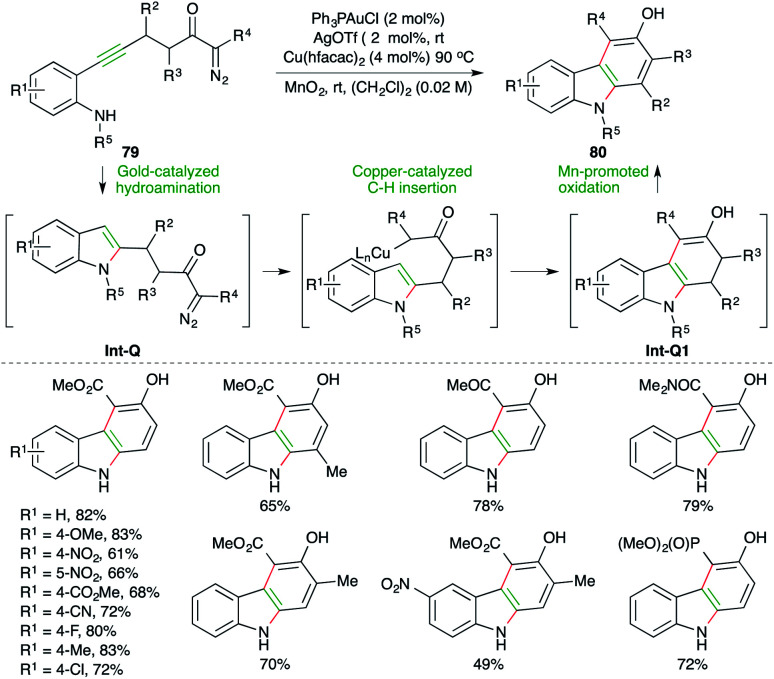
Hydroamination–C–H insertion cascade.

Carbazoles find themselves in a much specially focused area of organic synthesis as a result of their intriguing electrical, optical, and pharmacological properties, and as a result several strategies involving decorative synthesis upon indole have been reported. A gold-catalyzed carboamination reaction of alkynes for the synthesis of carbazoles has been reported by Park and co-workers. The reaction of diazo-tethered *o*-alkynyl anilines 79 with 2 mol% Ph_3_PAuCl and 2 mol% AgOTf, along with 4 mol% Cu(hfacac)_2_, furnished corresponding carbazoles 80 in good to excellent yield (Scheme 50).^64^ The reaction involves initial gold-catalyzed hydroamination reaction to form Int-Q, which upon subsequent copper-catalyzed C–H insertion gives Int-Q1. The Int-Q1 upon Mn-promoted oxidation leads to the formation of the desired carbazoles 80.

**Scheme 51:**
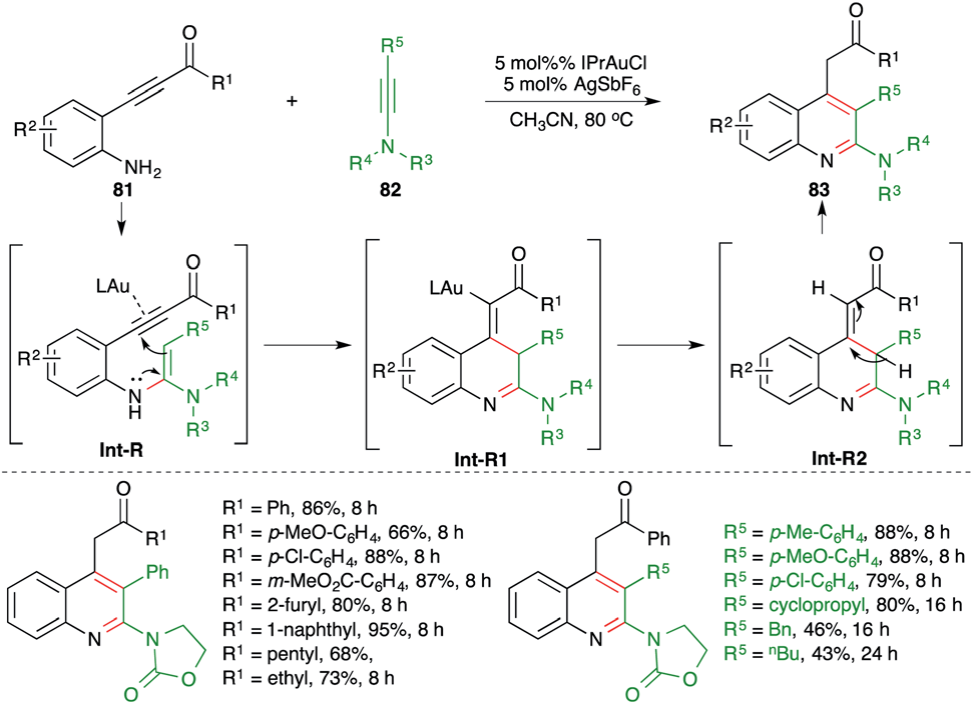
Synthesis of 2-amino quinolines using the carboamination of ynamides.

A gold-catalyzed carboamination cascade was studied by Arcadi and co-workers for the synthesis of 2-amino quinoline derivatives (Scheme 51).^65^ The reaction of *o*-alkynyl anilines 81 with ynamides 82 in the presence of 5 mol% IPrAuCl gave the corresponding 2-amino quinolines 83 in good yield. The reaction exhibited excellent scope and functional group compatibility. A range of ynamides were used in the reaction. The formation of the product can be explained as follows. Au-catalyzed intermolecular hydroamination furnishes enamines Int-R, which upon a subsequent intramolecular 5-*exo-dig* cyclization–protonation sequence afford Int-R2*via*Int-R1. Finally, proto-deauration of Int-R2 leads to the formation of 2-aminoquinolines.

**Scheme 52:**
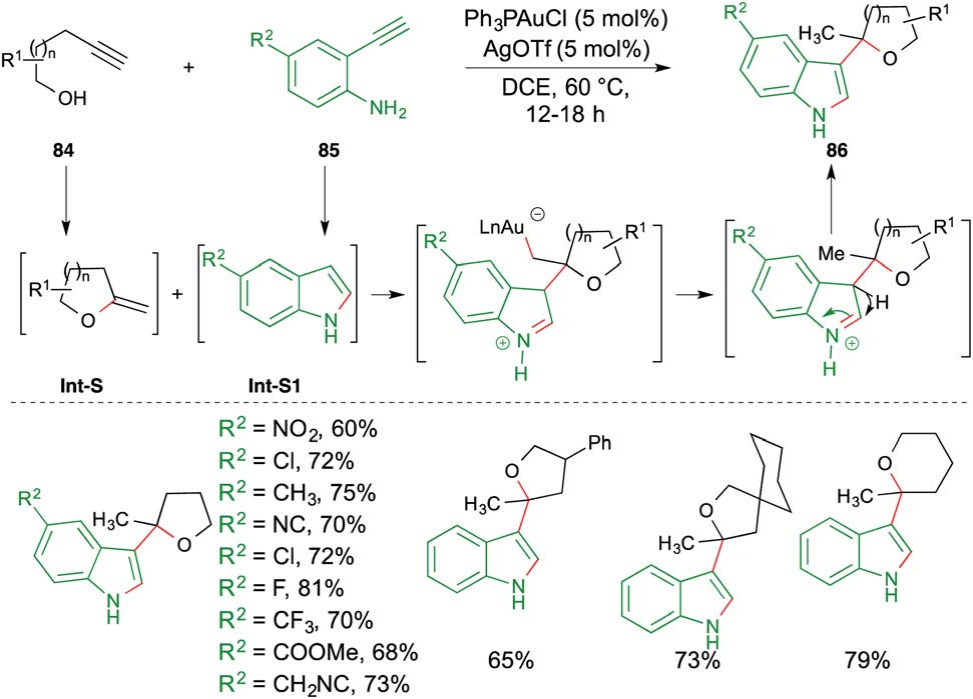
Hydroalkoxylation–carboamination cascade.

A carboamination reaction of terminal alkynols 84 and *o*-alkynyl anilines 85 was reported by Patil *et al.* for the rapid synthesis of variously C-3-substituted indoles 86. The reaction involved treatment of 84 with 85 in the presence of Ph_3_PAuCl (5 mol%) and AgOTf (5 mol%). The proposed mechanism consists of a tandem hydroalkoxylation–hydroamination–Friedel-Crafts alkylation cascade. Alkynols 84 and anilines 85 upon 5/6-*exo-dig* hydroalkoxylation and 5-*endo-dig* hydroamination give exocyclic enol ethers Int-S and indoles Int-S1, respectively. The indoles Int-S1 upon further Friedel–Crafts alkylation with Int-S furnish the required substituted indoles 86 in good yield (Scheme 52).^66^

**Scheme 53:**
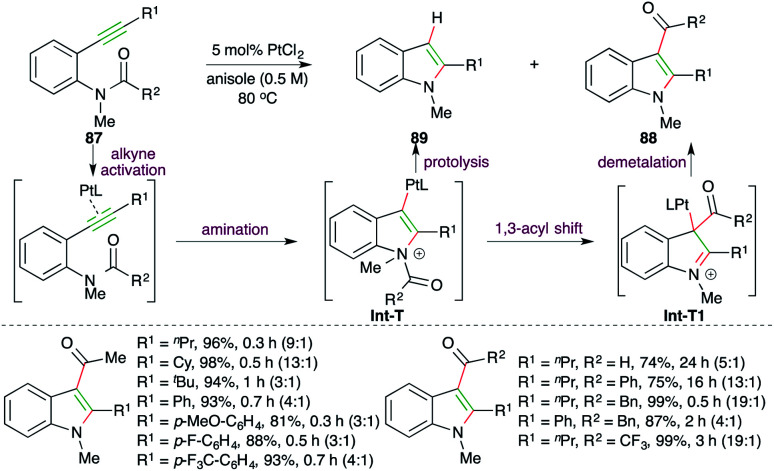
Carboamination accompanied by acyl migration.

A Pt-catalyzed intramolecular carboamination reaction was reported by Yamamoto and co-workers for the facile synthesis of indoles (Scheme 53).^67^ Reaction of *o*-alkynyl amides 87 with 1.5 mol% PtCl_2_ delivered the required 3-acyl indoles 88 as the major product along with indoles 89 as the minor product. The protocol involves Pt-catalyzed intramolecular amination of alkyne to generate Pt-tethered iminium ion Int-T, which upon subsequent 1,3-acyl migration affords Int-T1. Finally, proteolysis of Int-T1 furnishes the required indoles.

**Scheme 54:**
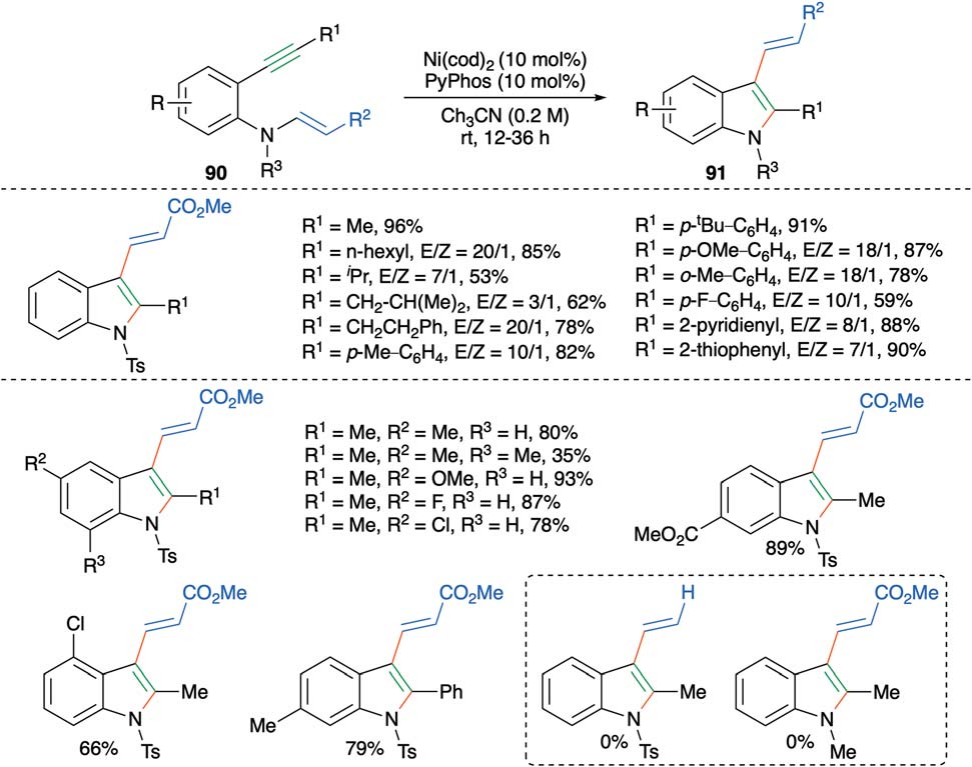
Synthesis of 3-allyl indoles using intramolecular carboamination.

Nickel-catalyzed *trans*-carboamination of internal alkynes was studied by Cho and co-workers for the facile synthesis of highly substituted indole motifs (Scheme 54).^68^ Reaction of various 2-alkynyl anilino acrylates 90 with 10 mol% each of Ni(cod)_2_ and PyPhos furnished the required indoles 91. Various indole derivatives were synthesized with good to excellent yield. It was observed that aromatic enamines (R^2^ = Ph) afforded the corresponding indoles in better yield and with better diastereoselectivity in comparison with aliphatic enamines (R^2^ = Ph).

**Scheme 55:**
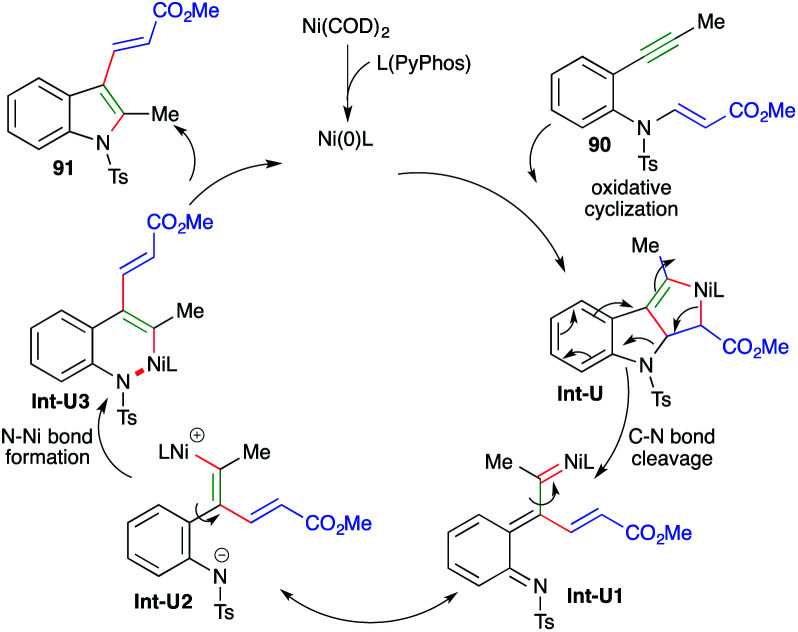
Reaction mechanism for the generation of 91.

The reaction mechanism involves oxidative cyclization of 90 to give nickelacycles Int-U, which upon C–N bond cleavage afford Int-U1. The Int-U1 upon charge redistribution leads to the formation of Int-U2. Int-U2 upon N–Ni bond formation gives Int-U3. Finally, reductive elimination of nickel furnishes the desired indole derivatives 91 (Scheme 55).

**Scheme 56:**
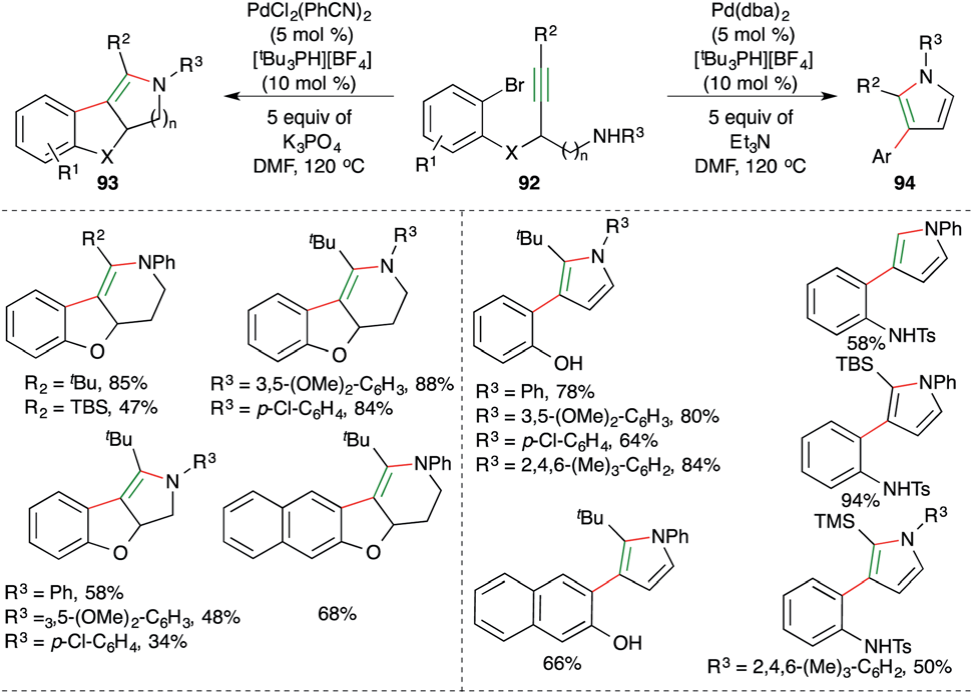
Pd-catalyzed *trans*-carboamination.

Werz *et al.* reported an elegant cascade consisting of intramolecular *anti*-carbopalladation–amination, *i.e.* carboamination for the diastereoselective synthesis of tetrasubstituted enamines and pyrroles (Scheme 56).^69^ They chose propargyl amine-tethered bromobenzenes 92 as suitable precursors, which upon treatment with PdCl_2_(PhCN)_2_ (5 mol%), [^*t*^Bu_3_PH][BF_4_] (10 mol%) and 5 equivalents of K_3_PO_4_ as the base, afforded tetrasubstituted amines 93 as the sole product in excellent yield. On the other hand, the use of 5 mol% Pd(dba)_2_ and 5 equivalents of Et_3_N as the base delivered pyrroles 94 as the sole product. Although various aliphatic alkynes and terminal alkynes were tried in the reaction to synthesize enamines/pyrroles, it was limited to aryl alkynes.

The reaction mechanism can be explained in two ways as follows.

**Scheme 57:**
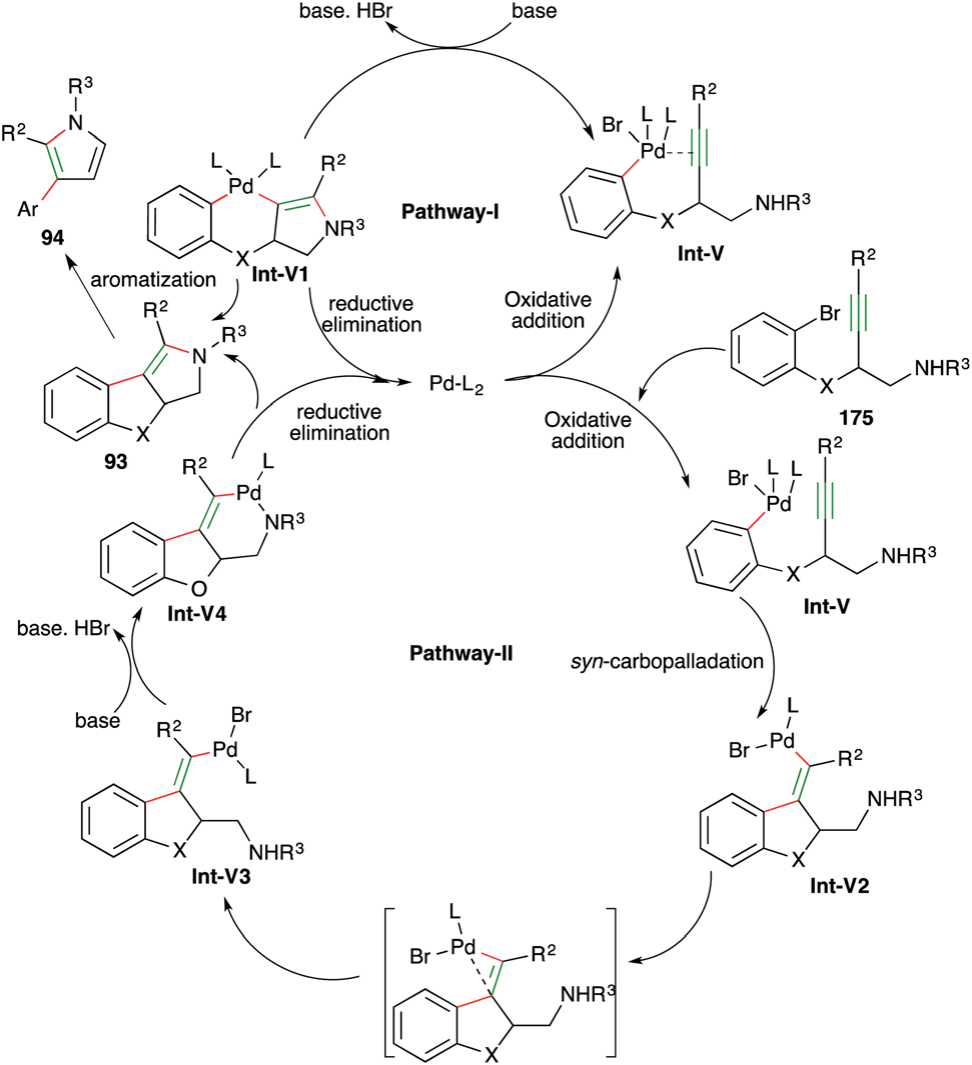
Reaction mechanism for obtaining 93 and 94.

In both pathways the initial step is oxidative addition of palladium to aryl bromide to give Int-V. After oxidative addition, in pathway-I, metal activates the alkynes and triggers the hydroamination step to form Int-V1, which upon subsequent reductive elimination furnishes enamines 93. The enamines upon elimination of phenol give pyrroles 94. In pathway-II, *syn*-carbopalladation occurs after the oxidative addition to give palladacycles Int-V2. The palladacycles then rotate to give *anti*-carbopalladation adducts Int-V3. The Int-V3 then undergo a second oxidative addition to furnish Int-V4, which upon reductive elimination give enamines 93 (Scheme 57).

**Scheme 58:**
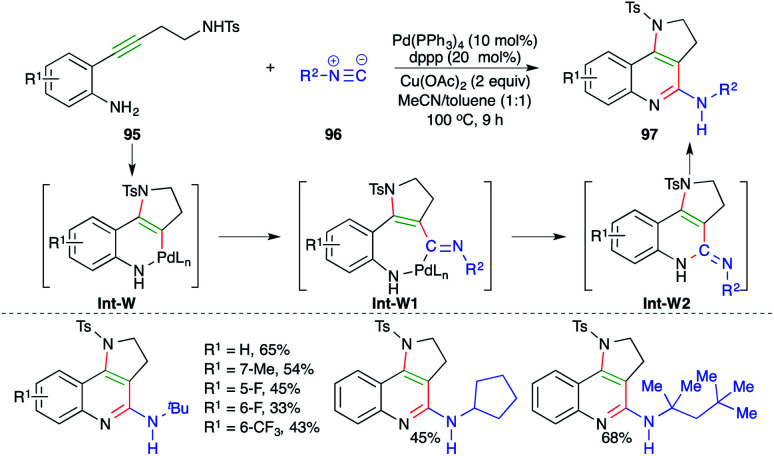
Synthesis of cyclic amine-fused quinolines using a carboamination approach.

A Pd-catalyzed carboamination cascade was described by Wu and co-workers for the facile synthesis of cyclic amine-fused-2-amino quinoline motifs (Scheme 58).^70^ The reaction of *o*-alkynyl anilines 95 with isocyanates 96 in the presence of 10 mol% Pd(PPh_3_)_4_ gave corresponding cyclic amine-fused-2-amino quinoline motifs 97 in good yield. The reaction mechanism involves initial *trans*-aminopalladation of 95 to generate enamines Int-W, which upon migratory insertion give Int-W1. Finally, Int-W1 upon reductive metalation and isomerization furnishes 97*via*Int-W2.

**Scheme 59:**
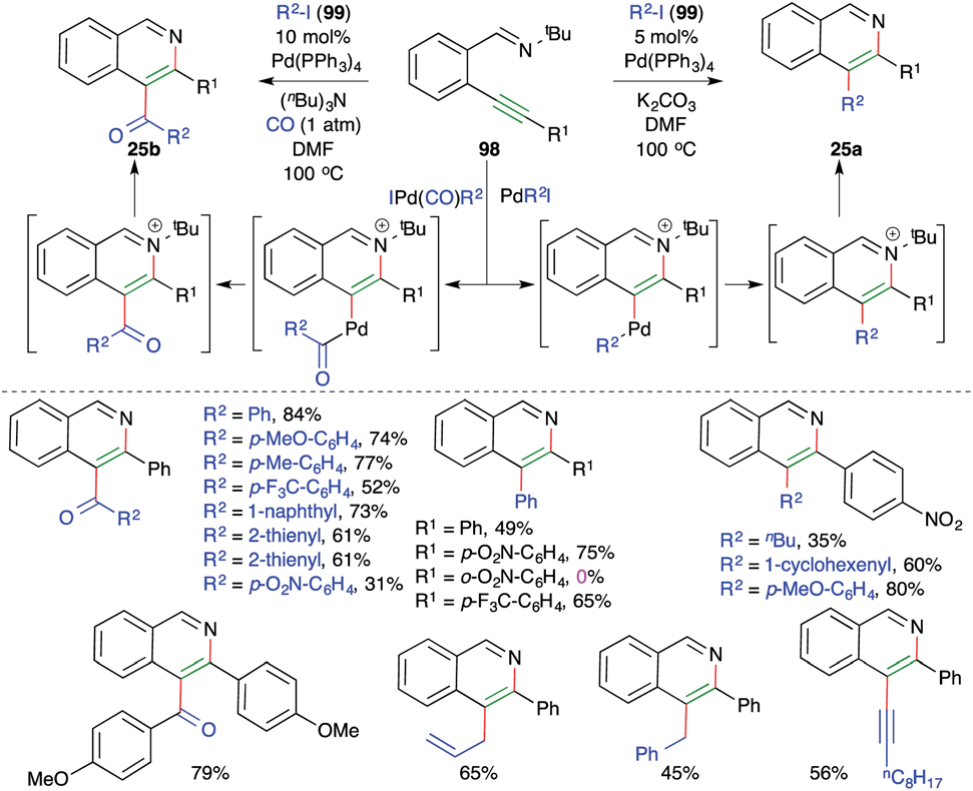
Synthesis of 3-acyl/aryl isoquinolines using a carboamination approach.

Activation of alkynes by alkylated/arylated/vinylated–Pd complex has emerged as an invincible technique for the concomitant construction of C–C and C–N/O/S bonds. In this context, Larock and co-workers have reported intermolecular carboamination for the synthesis of 3-arylated and 3-acylated isoquinolines. The reaction of alkyne-tethered imines 98 with aryl/vinyl/allyl/alkynyl iodides 99 in the presence of 5 mol% Pd(PPh_3_)_4_ gave the corresponding 3-substituted isoquinolines 25a. On other hand, when CO was added to the reaction medium, the corresponding 3-acylated isoquinolines 25b were obtained (Scheme 59).^71,72^

**Scheme 60:**
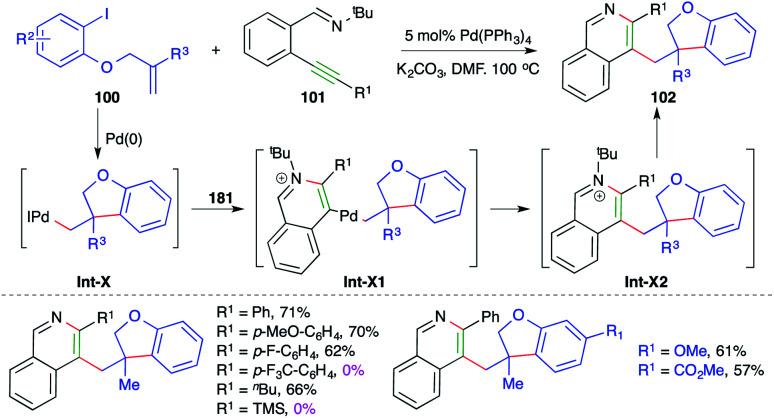
Tandem Heck reaction–carboamination approach.

Similarly, a Pd-catalyzed intermolecular carboamination cascade involving intramolecular amination of alkylated Pd-complex-activated alkynes was studied by Yao and co-workers for the quick assembly of 2,3-dialkylated isoquinolines. The reaction of *O*-allylated iodo phenols 100 with *o*-alkynyl aryl imines 101 in the presence of 5 mol% Pd(PPh_3_)_4_ gave the corresponding isoquinolines 102 in good yield (Scheme 60).^73^ The reaction has a very broad scope. Only internal alkynes were used; aryl alkynes gave better yields in comparison with aliphatic alkynes. The formation of the product can be explained as follows. The iodo phenol 101 gives alkylated-Pd complexes Int-X*via* intramolecular Heck cyclization, which in turn activate alkynes and to give Int-X1 through 6-*endo-dig* amination. The Int-X1 upon reductive elimination give Int-X2, which upon subsequent dealkylation give 102. Furthermore, the reaction did not work in the case of electron-deficient alkynes.

**Scheme 61:**
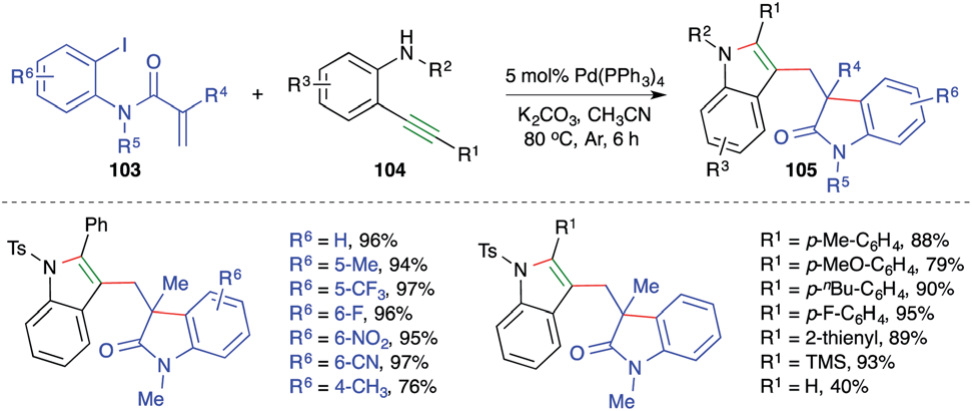
Tandem Heck reaction–carboamination approach.

On the lines of the previous report, a strategy with the same basis was studied by Lin and co-workers for the synthesis of bisindolyl methanes. The reaction of *o*-iodo/bromo aryl amides 103 with *o*-alkynyl amines 104 in the presence of 5 mol% Pd(PPh_3_)_4_ gave the corresponding bisindolyl methanes 105 in good yield (Scheme 61).^74^ The reaction offered rapid access to variously substituted bisindole moieties. Further, both internal and terminal/TMS-alkynes reacted in the desired manner with a little bit of discrepancy as terminal alkynes afforded the desired bisindoles in lesser yield. Despite the excellent functional group compatibility, free amides did not furnish the desired bisindoles.

**Scheme 62:**
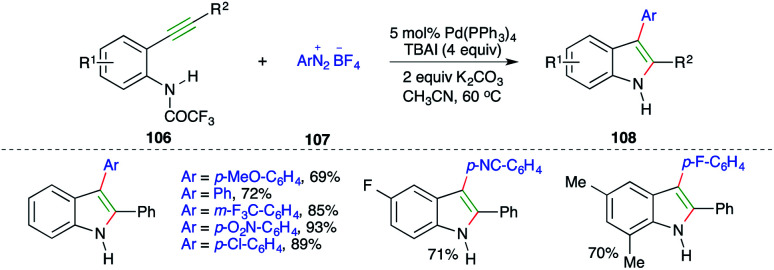
Use of arenediazonium tetrafluoroborates as carbon surrogates in carboamination.

Pd-catalyzed carboamination reaction using arenediazonium tetrafluoroborates as the carbon surrogate was explained in 2010 by Cacchi and co-workers. The reaction of *o*-alkynyltrifluoroacetanilides 106 with amines 107 in the presence of Pd(PPh_3_)_4_ and TBAI gave the corresponding indoles 108 in good yield (Scheme 62).^75^

**Scheme 63:**
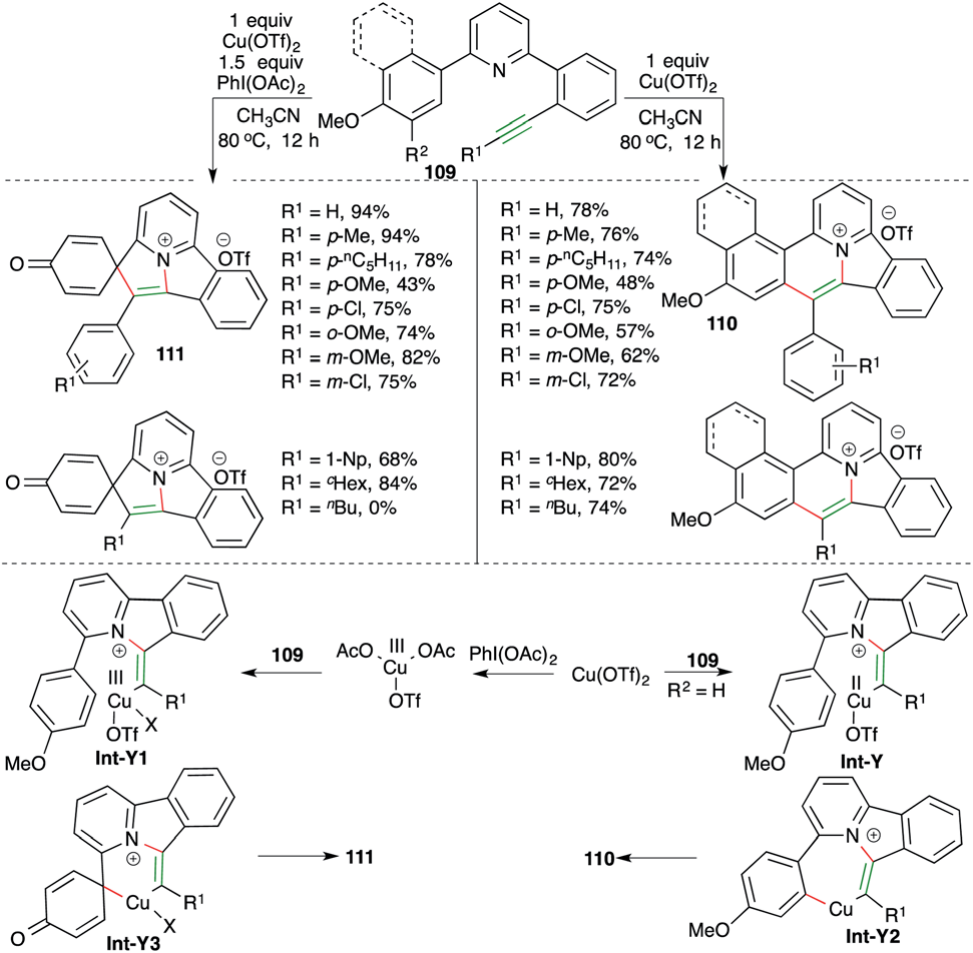
Intramolecular carboamination reaction for the synthesis of carbazoles.

Nitrogen-doped polycyclic aromatic hydrocarbons (PAHs) are useful motifs because of their application in the fields of optoelectronics, light-emitting diodes, supercapacitors, and bioimaging. They were recently employed as efficient DNA intercalators as well. In this direction, Patil and co-workers have reported a cascade consisting of intramolecular carboamination of alkynes using Cu-catalysis. Further, the divergent outcome of the current method could manifold its synthetic utility. The reaction of various alkyne-tethered pyridines 109 with Cu(OTf)_2_ gave corresponding PAHs 110 in good yield. Interestingly, the use of oxidant PhI(OAc)_2_ as an additive offered a completely different product 111 (Scheme 63).^76^ The reaction offered access to variously substituted PAHs. Interestingly, aliphatic alkynes did not produce the desired 111, but 110 was obtained in good yield. The detailed reaction mechanism is explained as follows. Alkynes 109 upon treatment with copper triflate give Int-Y or Int-Y1. Int-Y upon C–H insertion furnishes Int-Y2, whereas Int-Y1 gives Int-Y3*via ipso*-substitution. Finally, Int-Y2 and Int-Y3 give the desired PAHs 110 and 111 through reductive elimination.

**Scheme 64:**
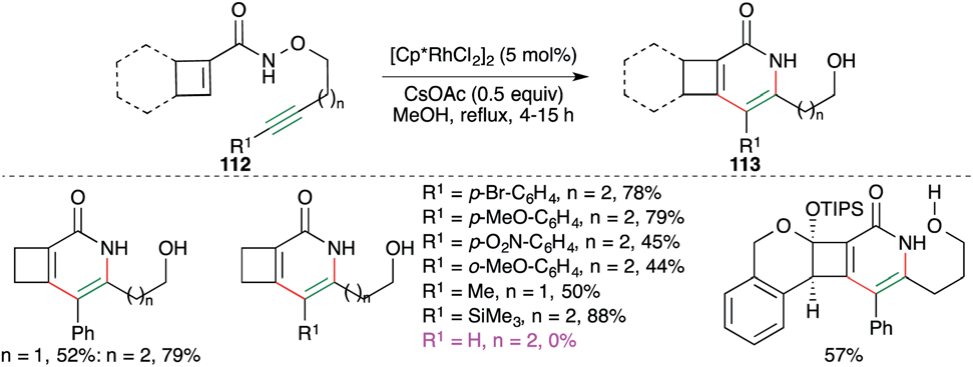
Tandem carboamination and N–O bond cleavage.

Cossy and co-workers have studied Rh-catalyzed intramolecular carboamination reaction of cyclobutene-tethered carboxamides 112 for the quick synthesis of cyclobuta[*c*]pyridones and -pyridines 113 (Scheme 64).^77^ The reaction worked well for internal as well as terminal alkynes. Furthermore, aliphatic, aromatic, and TMS-alkynes were also employed in the reaction to synthesize corresponding N-heterocycles. To manifold the synthetic utility, some complex N-heterocycles were also synthesized using the developed method.

**Scheme 65:**
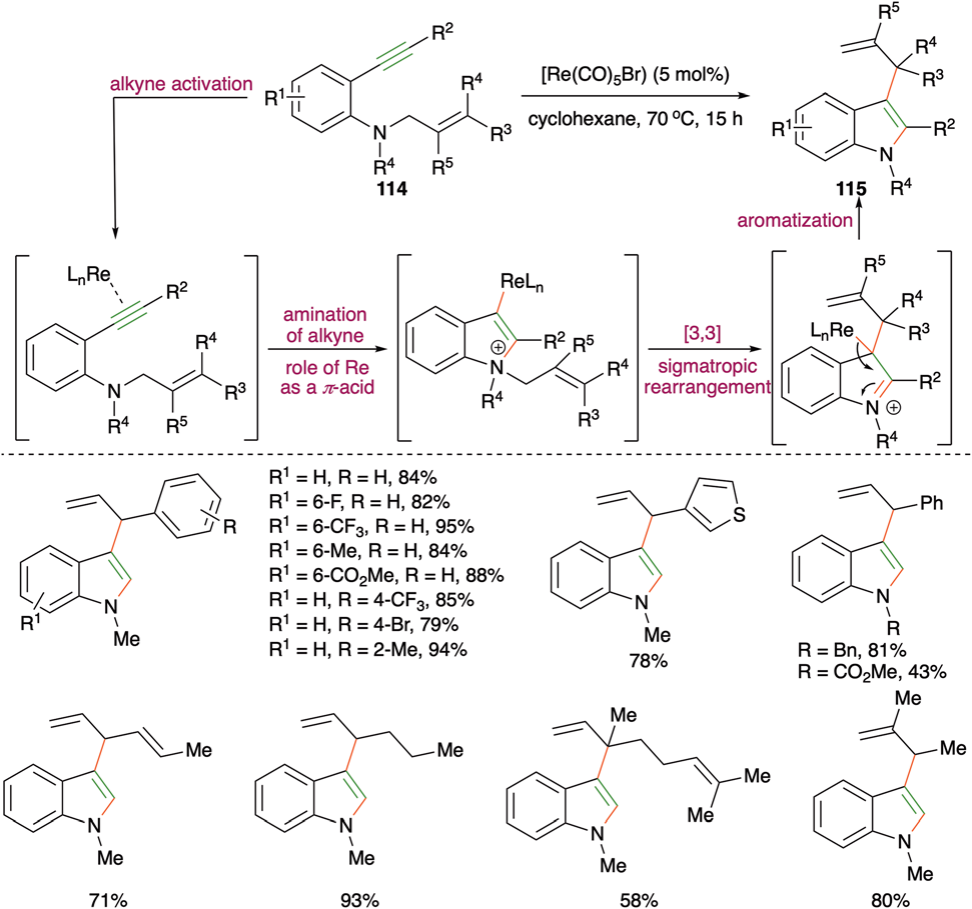
Rhenium-catalyzed intramolecular carboamination.

Rhenium-catalyzed carboamination of *o*-alkynyl *N*-homoallyl anilines 114 for the expedient synthesis of various 3-allyl indoles 115 was studied by Zi and co-workers (Scheme 65).^78^ It is not the first time they had synthesized such reported scaffolds, but here they could successfully replace the use of precious metals such as Pd, Pt, and Rh with rhenium (Re). Various 3-allyl indoles were synthesized using the developed method. Moreover, they have also used this method for the synthesis of 3-allylated furans. The proposed mechanism suggested that the formation of 3-allylated indoles takes place *via* initial rhenium activation of the alkynes by Re playing the role of a π-acid, followed by a charge-accelerated [3,3]-sigmatropic rearrangement.

## 4. Metalla-electrocatalyzed carboamination

Indubitably, transition metal-catalyzed carboamination of alkynes has solved many long-standing problems and has offered a plethora of strategies for the synthesis of various N-bearing scaffolds and their congeners. Although it has drastically reduced the catalyst loading, the stochiometric use of co-oxidants varying from transition metals to hypervalent iodine has brought some non-negotiable limitations in terms of generation of a considerable amount of byproducts. To overcome these deficiencies, the synthetic community has chosen electrolysis. Arguably, metalla-electrocatalyzed carboaminations have solved the stochiometric use of co-oxidants and have delivered many elegant methods. A recent account describing developments in this context was documented in 2019 by Ackermann.^79^ We wish to include the preceding developments in this review to position the efficacy of the current strategy in this field.

**Scheme 66:**
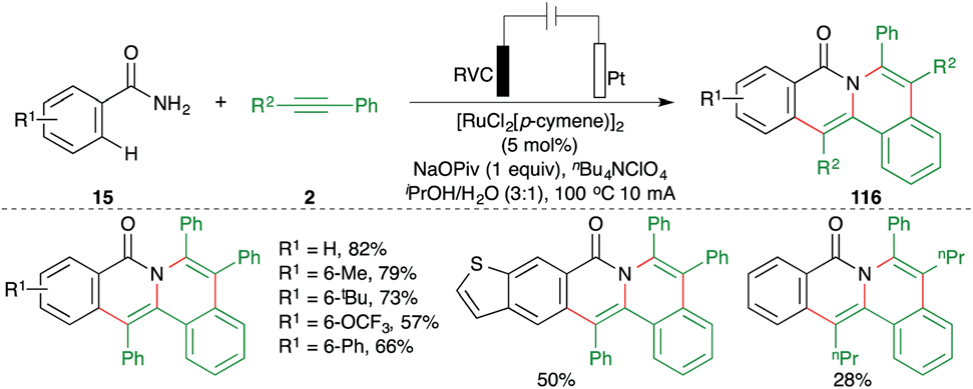
Bis-carboamination approach.

An electrochemically enabled, Ru-catalyzed bis-carboamination of aryl amides was developed by Tang and co-workers for the synthesis of polycyclic isoquinolone motifs. The method included the reaction of aryl amides 15 with alkynes 2 in an electrolytic cell in the presence of 5 mol% [RuCl_2_(*p*-cymene)]_2_ to give the corresponding isoquinolones 116 in good yield. The key feature of the reaction was the observed regioselectivity in the case of unsymmetrical alkynes (Scheme 66).^80^

**Scheme 67:**
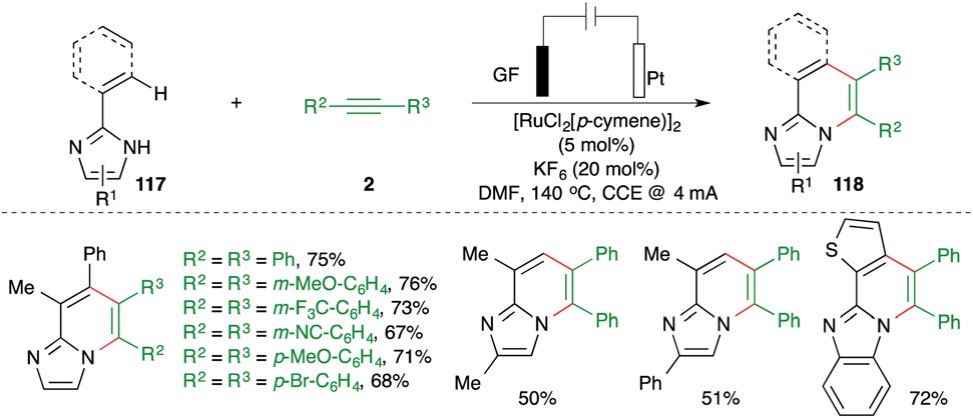
Ru-catalyzed carboamination in an undivided cell.

Ackermann and co-workers have reported carboamination of alkenyl imidazoles 117 with various alkynes 2 using an operationally simple undivided cell setup equipped with a GF (graphite felt) anode and a Pt cathode and 5 mol% [RuCl_2_(*p*-cymene)]_2_ as the metal catalyst for the synthesis of N-heterocycles 118 (Scheme 67).^81^ The isolated aza–ruthenium complex and its crystal structure revealed formation of a ruthenacycle through the C–H insertion, which after carbo-ruthenation of alkynes and reductive metalation led to the formation of the desired adducts.

**Scheme 68:**
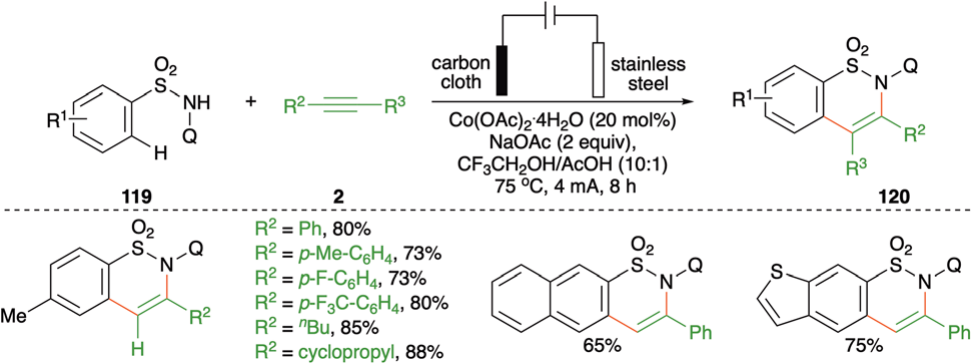
Co-catalyzed carboamination in an undivided cell.

On similar lines, a Co-catalyzed electrochemical carboamination reaction of directing group-tethered (quinolin-8-yl) aryl sulfonamides was elaborated by Lei and co-workers. The developed method involved treatment of aryl amides 119 with alkynes 2 in an electrochemical cell containing 5 mol% cobalt salt to give the corresponding sultams 120 in good yield (Scheme 68).^82^ Only terminal alkynes were used extensively as internal alkynes led to the formation of a mixture of regio-isomers.

## 5. Transition-metal-free carboamination

**Scheme 69:**
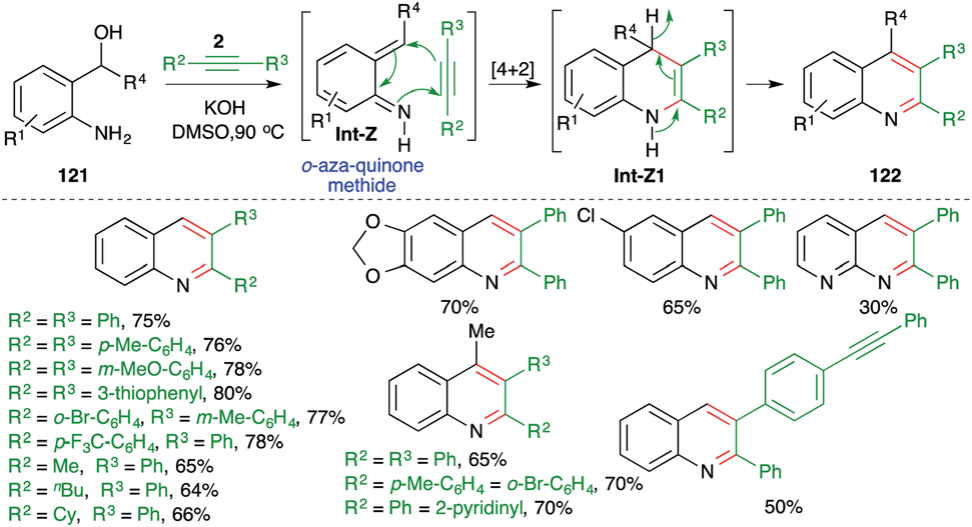
Base-promoted carboamination of alkynes.

Undoubtably, *o*-quinone methide has been used extensively as the diene partner for the synthesis of oxygen-bearing heterocycles. In contrast, its nitrogen congener, *o*-aza-quinone methide, is comparatively much less studied.^83^ In this domain, methods describing intermolecular [4 + 2] cycloaddition of *o*-aza-quinone methide with alkynes have been studied for the synthesis of quinolines. In this direction, Verma and co-workers have reported a metal-free base-promoted intermolecular carboamination strategy for the synthesis of variously substituted quinolines (Scheme 69).^84^ The method involved treatment of *o*-amino benzyl alcohols 121 with internal alkynes 2 in the presence of 1 equiv. of a base such as KOH to furnish quinolines 122 in good yield. The reaction involved the base-promoted generation of *o-aza* quinone methides Int-Z from 121, which upon subsequent intermolecular carboamination reaction with alkynes 2 furnished dihydroquinolines Int-Z1. Finally, autoxidation of Int-Z1 delivered variously substituted quinolines. Although reactions have employed aromatic alkynes (R^2^ = R^3^ = aryl) or aromatic aliphatic alkynes (R^2^ = alkyl, R^3^ = aryl), aliphatic alkynes (R^2^ = R^3^ = alkyl) did not furnish the required quinolines.

**Scheme 70:**
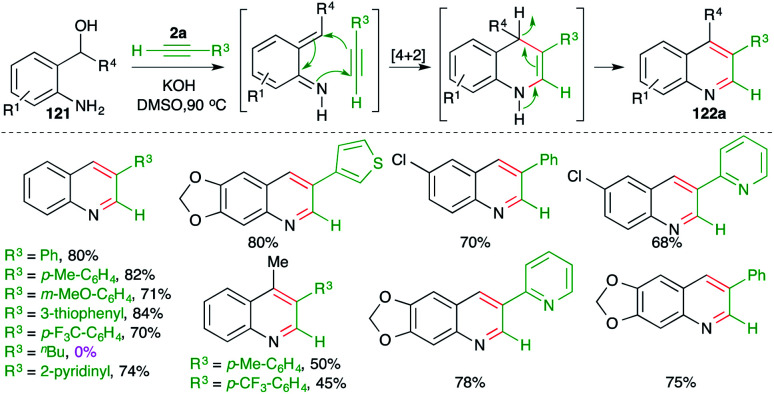
Base-promoted carboamination of alkynes.

In continuation of their study on the synthesis of quinolines, they also reported reaction of *o*-amino benzyl alcohols 121 with terminal alkynes 2a in the presence of 1 equiv. of a base such as KOH to furnish the requisite 3-substituted quinolines 122a in good yield (Scheme 70).^85^

**Scheme 71:**
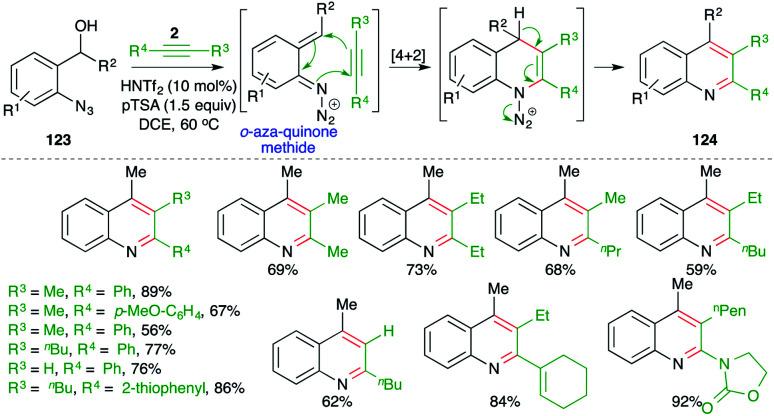
Synthesis of quinoline using a metal-free carboamination approach.

On similar lines, Niggemann and Stopka have studied metal-free, acid-promoted intermolecular carboamination of alkynes for the quick synthesis of variously substituted quinolines. The reaction of *o*-azido benzyl alcohols 123 with alkynes 2 in the presence of 1.5 equiv. of pTSA and 10 mol% triflimide furnished the requisite quinolines 124 in good yield (Scheme 71).^86^ The reaction has excellent substrate scope. Most types of alkynes participated in the reaction, leading to the formation of corresponding quinoline derivatives. As well as classical alkynes, enynes and ynamides were also used to synthesize their respective quinolines.

**Scheme 72:**
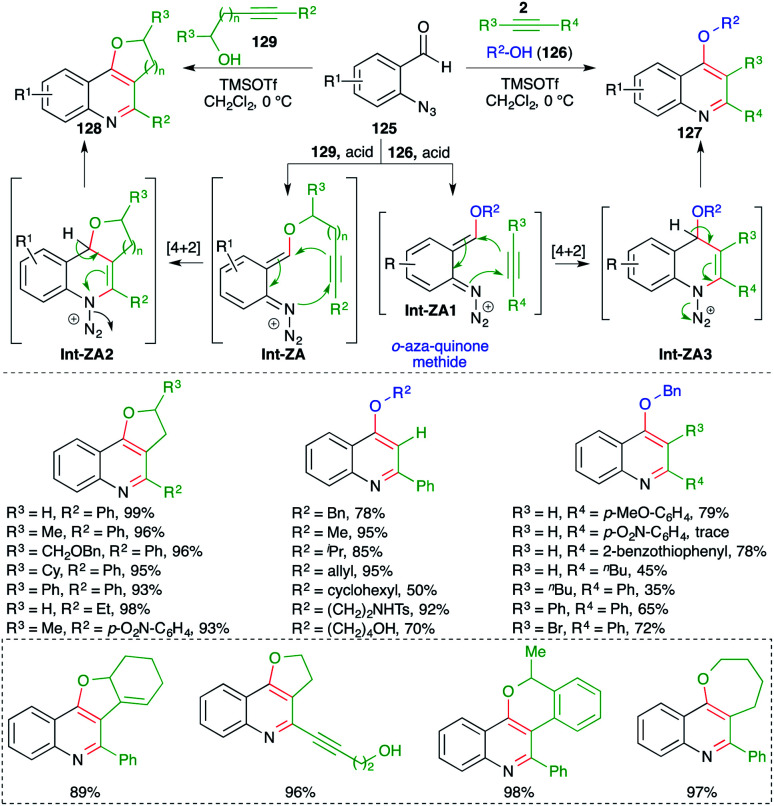
Synthesis of 4-alkoxy quinoline using a metal-free carboamination approach.

In contrast to the attention devoted towards quinoline, synthesis of its congeners such as 4-alkoxy quinolines as well as cyclic ether-fused quinolines has been less explored. In this context, Gharpure and co-workers reported a metal-free Lewis acid-promoted multicomponent carboamination cascade for the synthesis of these motifs. The method included the reaction of *o*-azido benzaldehydes 125 with alkynes 2 and alcohol 126 in the presence of TMSOTf as Lewis acid to generate 4-alkoxy quinoline 127 in excellent yield. The developed method has a very broad substrate scope with excellent functional group tolerance. Almost all types of alkynes participated to give the corresponding 4-alkoxy quinolines, except electron-deficient alkynes *i.e.* aryl alkynes with an electron-withdrawing group on the aromatic ring (Scheme 72).^87^ Further, the method was also applied in the synthesis of cyclic ether-fused quinolines 128 from the corresponding alkynols 129 and *o*-azido benzaldehydes 125. The formation of desired quinolines can be explained as follows. The reaction of *o*-azido benzaldehydes 125 with alcohol 129, 126 generated *o-aza* quinone methide Int-ZA-ZA1 through acid-mediated condensation. The Int-ZA-ZA1 then underwent inter- or intramolecular carboamination reaction *i.e.* a formal [4 + 2] cycloaddition reaction leading to the formation of dihydroquinoline Int-ZA2-ZA3, which upon concomitant elimination of H and N_2_ afforded desired quinolines 128 and 127, respectively.

**Scheme 73:**
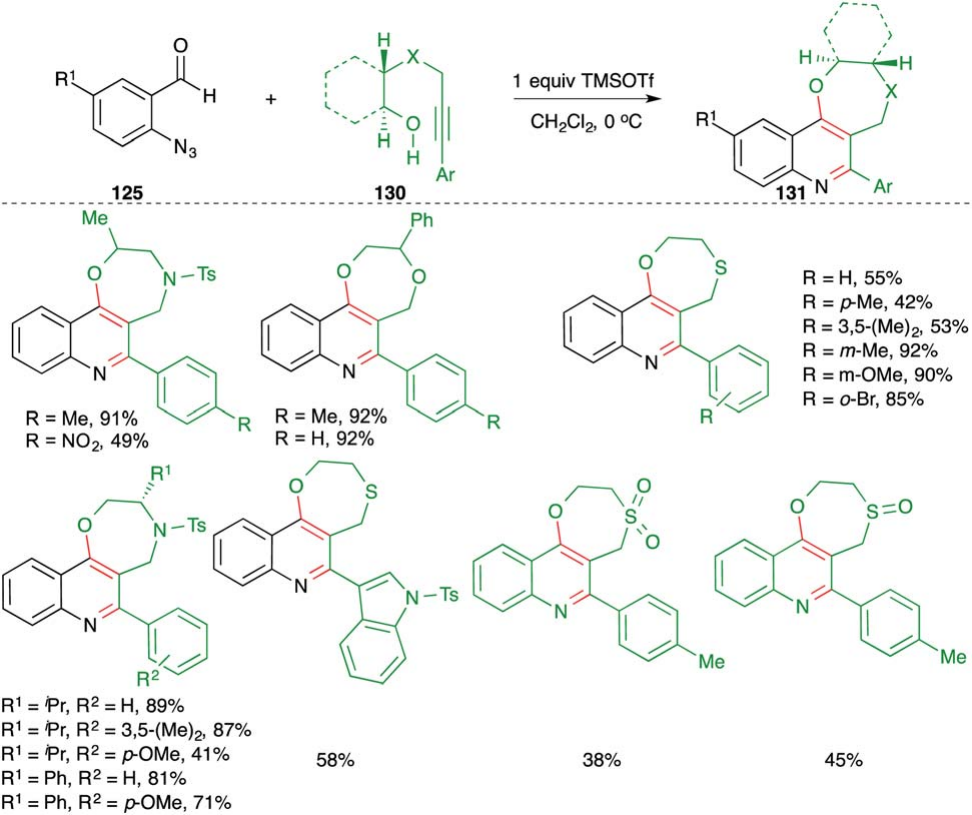
Synthesis of cyclic ether-fused quinoline using a metal-free carboamination approach.

On similar lines, they also applied the developed strategy for the synthesis of 1,4-heterocycle-fused quinoline motifs 130 from hetero-atom tethered alkynols 131 (Scheme 73).^88^ Various enantiopure 1,4-oxazepino-quinolines were synthesized in good yield from the corresponding amino acid-derived alkynols. Further, *O*/*S*-tethered alkynols were employed in the reaction to synthesize dioxepino/oxathiepino-quinolines.

**Scheme 74:**
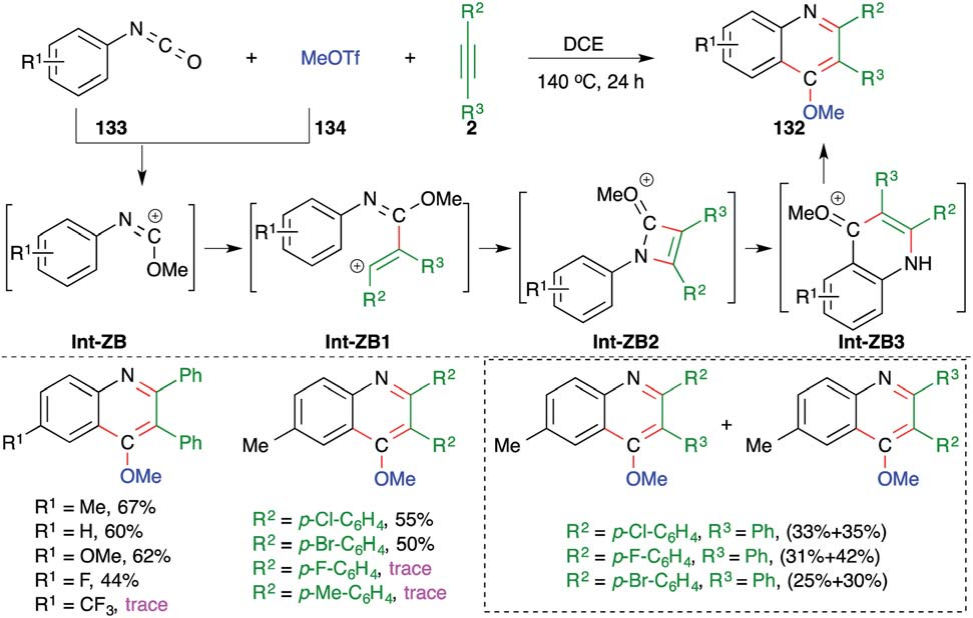
Synthesis of 4-methoxy quinoline using a metal-free carboamination approach.

On similar lines, 4-methoxy quinolines 132 were synthesized under metal-free conditions from the intermolecular carboamination of aryl isocyanates 133 with alkynes 2 and MeOTf 134 (Scheme 74).^89^ Various aryl isocyanates and alkynes were employed in the reaction to give the corresponding quinolines. However, the reaction is limited to only aryl alkynes, and in the case of unsymmetrical alkynes, a mixture of separable regio-isomers was obtained. The proposed reaction mechanism involved the initial generation of carbocation Int-ZB from 133 and methyl triflate, which upon intermolecular trapping with alkyne led to the formation of a new vinyl cation Int-ZB1. The Int-ZB1 upon intramolecular amination gave Int-ZB2, which upon ring opening furnished Int-ZB3. Finally, intra-molecular Friedel–Crafts alkylation afforded 132.

**Scheme 75:**
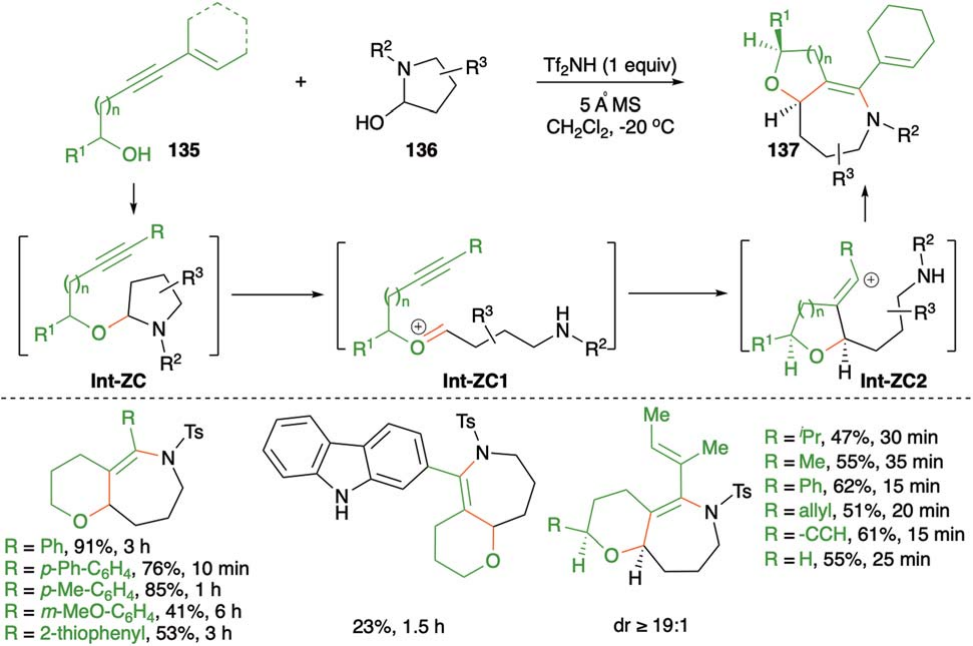
Synthesis of bicyclic heterocycles using a metal-free carboamination approach.

An elegant approach describing metal-free, intermolecular carboamination reaction of alkynols 135 with cyclic aminals 136 was described by Frontier and co-workers for the stereoselective synthesis of cyclic ether-fused cyclic enamines 137 (Scheme 75).^90^

The suggested reaction mechanism involves formation of aminals Int-ZC through the coupling of alkynols 135 with aminals 136*via* iminium ion trapping. The aminals Int-ZC give oxonium ions Int-ZC1 and vinyl cations Int-ZC2, respectively, through a ring-opening-alkyne trapping cascade. Finally, Int-ZC2 upon intramolecular amination leads to the formation of 137. Various bicyclic heterocycles were prepared in good yield and with good diastereoselectivity.

**Scheme 76:**
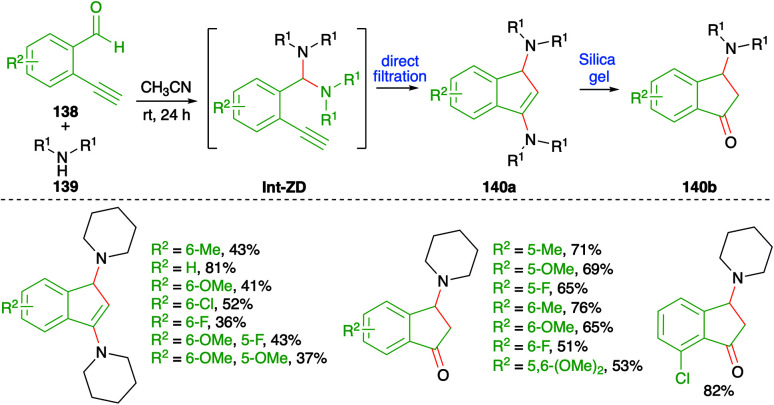
Synthesis of indanones using a metal-free carboamination approach.

An iminium ion-driven intermolecular carboamination strategy was depicted by Wong *et al.* for the facile synthesis of amino indenes 140a and 3-amino indanones 140b (Scheme 76).^91^ The method involved reaction of *o*-alkynyl benzaldehyde derivatives 138 with secondary amines 139 in acetonitrile to give 140a as the sole product through direct filtration of crude mass. Further, silica gel column chromatography of the crude reaction mixture gave 140b through hydrolysis of 140a. The reaction involves the initial formation of aminal Int-ZD, which upon intramolecular cyloisomerization leads to the formation of 140a. Finally, quick passage of 140a furnishes 3-amino indanones 140b in good yield. The reaction has an excellent substrate scope and good functional group tolerance. However, the reaction is limited to terminal alkynes only.

**Scheme 77:**
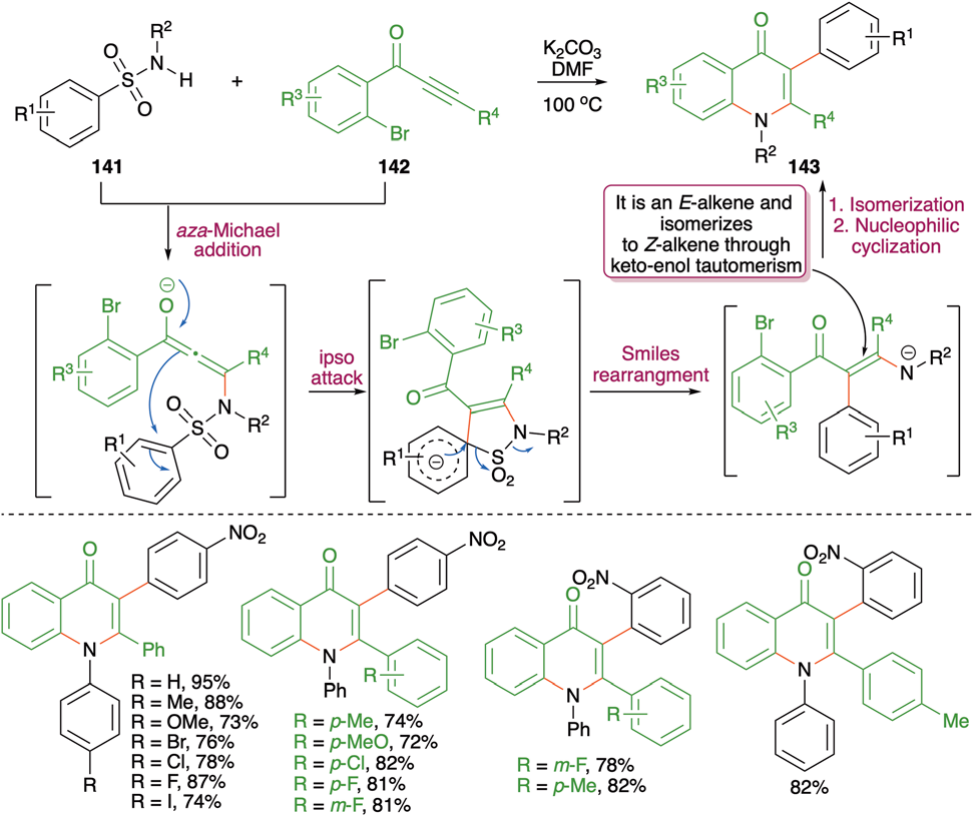
Metal-free *aza*-Michael addition–Smiles rearrangement sequence.

A metal-free base-promoted intermolecular carboamination reaction of amides 141 with *o*-bromoaryl ketones 142 was exemplified by Ma and co-workers for the rapid construction of 4-quinoline moieties 143 (Scheme 77).^92^ Potassium carbonate was used as base in DMF as solvent. The reaction has indeed a very wide substrate scope. The carboamination involved a base-promoted *aza*-Michael addition–Smiles rearrangement–SO_2_-extrusion–intramolecular aromatic nucleophilic substitution cascade to arrive at the desired quinolones. The method also worked well in the case of aliphatic alkynones (R^4^ = methyl).

**Scheme 78:**
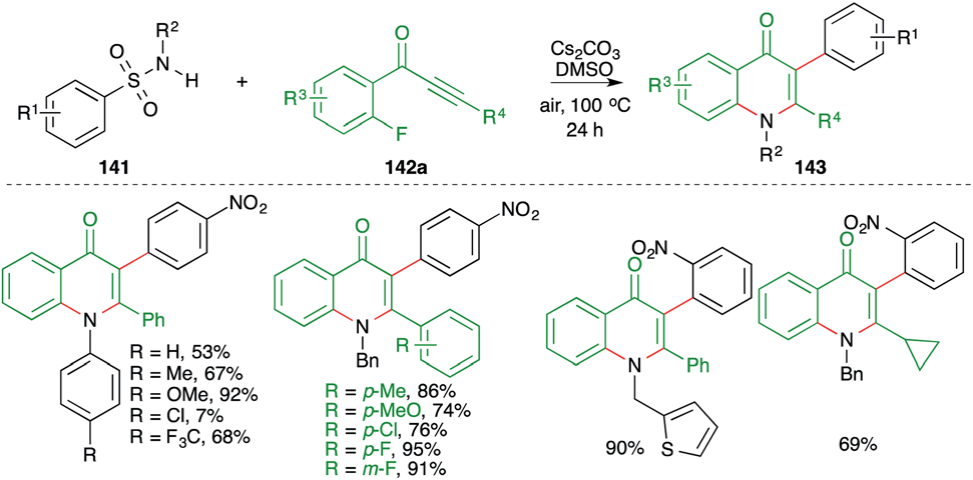
Synthesis of quinolones using a carboamination reaction.

On similar lines, a green approach was developed by Cheng and co-workers for the synthesis of 4-quinolones. They used similar precursors (fluorides instead of bromides) to arrive at 4-quinolones through the use of Cs_2_CO_3_ in air instead of K_2_CO_3_ under nitrogen atmosphere (Scheme 78).^93^ Further, the method has a very wide substrate scope. However, only a small number of examples were accomplished using aliphatic alkynones.

**Scheme 79:**
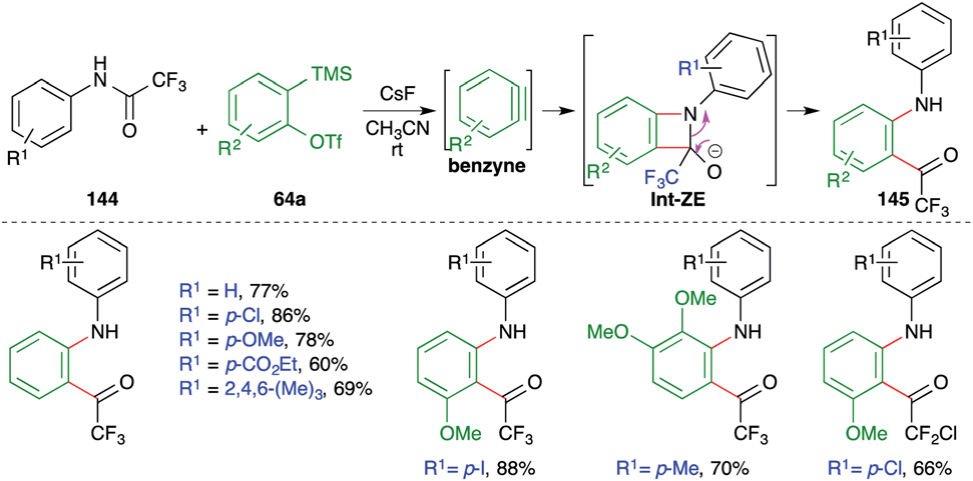
Metal-free carboamination of benzynes.

A metal-free intermolecular carboamination approach was reported by Larock and co-workers for the facile synthesis of *o*-acyl anilines (Scheme 79).^94^ Reaction of *N*-aryltrifluoroacetamides 144 with 2-(trimethylsilyl)aryl triflates 64a in the presence of CsF gave *o*-acyl anilines 145 in good yield. The fluoride accelerates the formation of benzyne, which upon either [2 + 2] cycloaddition with imine or step-wise nucleophilic attack forms oxetane derivatives Int-ZE. The Int-ZE upon ring-opening and protonation leads to the formation of *o*-acyl anilines.

## 6. Summary and outlook

In this review, different strategies to conduct the intermolecular/intramolecular carboamination of alkynes leading to the synthesis of various chemically interesting scaffolds have been discussed. In this context, events utilizing both metal and metal-free conditions were covered.

Further, the carboamination of alkynes offered an array of excellent approaches for the synthesis of nitrogen-bearing aromatic heterocycles. These developed methods were studied under both metal and metal-free conditions. In particular, the metal-catalyzed transformations were broadly classified into two categories. In category 1, the key step of the transformation was either metal-catalyzed decarbonylation or decarboxylation or denitrogenation, and this was followed by an alkyne insertion–reductive elimination sequence. In category 2, the reactions were driven by directing group-assisted/free C–H bond functionalization leading to the formation of a metallacycle, which, upon alkyne insertion and reductive elimination, offered access to N-bearing aromatic entities. Further, metal-catalyzed cycloaddition reactions were also used in the carboamination reactions. On the other hand, the corresponding metal-free approaches have offered some excellent paths towards the synthesis of N-bearing complex entities.

Although significant progress has been achieved in the field of the carboamination of alkynes, still there is room for practical developments. Most of the developed strategies rely upon the use of costly transition metals, leaving a blank space for corresponding metal-free approaches. Further, methods utilizing the metal-based as well as metal-free carboamination of alkynes have led to the documentation of some excellent protocols for the rapid synthesis of N-bearing aromatic heterocycles. However, the use of alkynes was found to be limited and in most of the cases, an excess of alkynes was also used. One more key understudied area is that, in the case of unsymmetrical alkynes, a mixture of regio-isomers was obtained. Although Ackermann's group have solved this problem to a major extent, further development of methods using unsymmetrical alkynes leading to the formation of single regio-isomers is highly desirable. In order to avoid the use of costly metals, various groups across the globe have invented a unique strategy, *i.e.*, metalla-electrocatalyzed carboamination. Although it has solved cost-effectiveness problems, the regioselectivity issue still persists. Though the above functionalization has offered some really good access to unsaturated heterocycles, methods for the synthesis of the corresponding saturated heterocycles are scarce in the literature, despite the presence of these heterocycles in a wide range of bioactive natural products. Thus, a unified effort to tackle these issues is exceedingly required so that these methods can be applied in the synthesis of various natural products and pharmacophores.

Apart from the above-mentioned deficiencies, one of the most important concerns is the use of terminal alkynes in the carboamination reactions. The formation of metal salts with alkynes having acidic hydrogen may be one cause of the unsuitability in this type of reaction. However, in some cases they have been used but found to be unsatisfactory. Thus, a general and concise approach supporting the use of terminal alkynes is desirable.

## Conflicts of interest

There are no conflicts to declare.

## Supplementary Material
